# Sex‐Dependent Effects of Angiotensin II and Calcineurin in the Vasculature of Mice

**DOI:** 10.1111/apha.70213

**Published:** 2026-03-27

**Authors:** Alexander Nolze, Sindy Rabe, Stefanie Ruhs, Nicole Strätz, Katja Quarch, Conny Köhler, Claudia Grossmann

**Affiliations:** ^1^ Julius Bernstein Institute of Physiology Martin Luther University Halle‐Wittenberg Halle (Saale) Germany

**Keywords:** angiotensin II, calcineurin, cardiovascular diseases, hypertension

## Abstract

**Aim:**

Cardiovascular diseases display strong sex differences. Angiotensin II (AngII) is implicated in this process. The ubiquitously expressed enzymatic beta subunit of calcineurin (PPP3CB), a serine/threonine phosphatase, can mediate pathological effects of AngII in the heart. Our aim was to explore the role of calcineurin in sex‐dependent AngII‐mediated vascular changes.

**Methods:**

We used female and male mice with a global PPP3CB knockout that were treated with AngII for 4 weeks as an in vivo model. For validation experiments and investigation of signaling pathways, primary aortic vascular smooth muscle cells (aVSMCs) isolated from respective female and male WT mice were utilized.

**Results:**

AngII‐induced increase in blood pressure was less pronounced and not calcineurin‐dependent in female compared to male mice with no changes in media thickness or lumen area. Wire and pressure myography showed an AngII‐induced calcineurin‐dependent endothelial dysfunction in males but not in females. In aVSMCs from female mice, AngII did not influence wound closure or cell proliferation as was detectable in aVSMCs of male mice. As an underlying mechanism for these sex differences in long‐term AngII effects, RNA‐seq data and IPA revealed differentially regulated genes and pathways, involving extracellular matrix components, calcineurin, *Ctgf*, *Egfr*, and *Tgfb1*. Downstream of *Egfr,* we identified sex‐dependent activation of PKC signaling in male and ERK/MAPK signaling in female as mediators of *Ctgf* expression.

**Conclusion:**

Overall, the relevance of AngII‐calcineurin signaling for pathophysiological effects in the vasculature differs between female and male mice, suggesting both sexes require customized prevention and treatment strategies for cardiovascular disorders.

**Abbreviations**
AngII: angiotensin II
Egf: epidermal growth factor
Egfr: epidermal growth factor receptor
FCS: fetal calf serum
IPA: ingenuity pathway analysis
KO: knockout
Postn: periostin
PPP3CB: catalytic beta subunit of calcineurin
SBP: systolic blood pressure
Tgfb1: transforming growth factor beta 1
VMSCs: vascular smooth muscle cells
WT: wildtype

## 1 Introduction

Cardiovascular diseases belong to the aging‐related diseases and are the main cause of death in the industrialized world. They are characterized by organ dysfunction, inflammation, fibrosis, and hypertrophy [1, 2, 3, 4]. During these processes, the composition of blood vessels changes due to increased proliferation of vascular smooth muscle cells (VSMCs) or an increased secretion of extracellular matrix components [5]. Angiotensin II (AngII), as part of the renin‐angiotensin‐aldosterone‐system (RAAS), is a well‐known mediator of such alterations and is known for displaying sex‐specific differences [6, 7].

Besides activating PLC, MAP kinases, and increasing [Ca^2+^]_intracellular_, AngII is able to activate the serine/threonine phosphatase calcineurin (PPP3) and thereby mediate pathological effects of the RAAS in the cardiovascular system [8, 9, 10]. Calcineurin is a heterodimer consisting of a regulatory and a catalytic subunit, which is activated by Ca^2+^‐calmodulin complexes. Once activated, calcineurin dephosphorylates transcription factors like NFaT or CREB to regulate gene transcription [11, 12, 13]. There are three isoforms of the catalytic subunit, PPP3CA, PPP3CB and PPP3CC, of which the former two are expressed ubiquitously. Mice with a global knockout of only PPP3CB develop normal into adulthood and are protected from increase in heart size after isoprenaline infusion in comparison to WT animals [8].

Previously, we found in male WT mice, that AngII infusion for 4 weeks increases systolic blood pressure up to 149.9 ± 3.9 mmHg, and that this effect is attenuated in animals with a knockout of the catalytic beta subunit of calcineurin (PPP3CB KO) [14]. Additionally, aortic remodeling with increased aortic media thickness and lumen area was found in WT but not in PPP3CB KO mice after AngII stimulation. With gene set enrichment analysis of RNA‐Seq data and qPCR validation experiments, we found that AngII via PPP3CB leads to an increase in extracellular matrix (ECM) components like collagen1a1, collagen3a1, collagen4a1, fibronectin, periostin, and osteonectin. Likewise, wound closure of primary aVSMCs in a scratch assay was accelerated by AngII only in the presence of PPP3CB. As the underlying signaling cascade leading to pathological AngII effects in VSMCs of male mice, we identified AT_1_R‐PPP3C‐EGFR‐TGFB1‐CTGF signaling. Our results suggest that calcineurin plays a crucial role in pathological AngII effects in aorta and aVSMCs, suggesting that inhibition of calcineurin is beneficial for preventing or treating vascular diseases. Gender and sex differences in cardiovascular diseases and also in AngII signaling have been reported but are not well characterized or understood [15, 16]. Therefore, we now investigated the effect of long‐term AngII application on the vasculature of WT and PPP3CB female mice and compared the results to our previous findings in their male littermates [14].

## 2 Results

### 2.1 Long‐Term AngII Treatment Leads to a Mild Increase in Systemic Blood Pressure in Female WT and PPP3CB KO Mice

**FIGURE 1:**
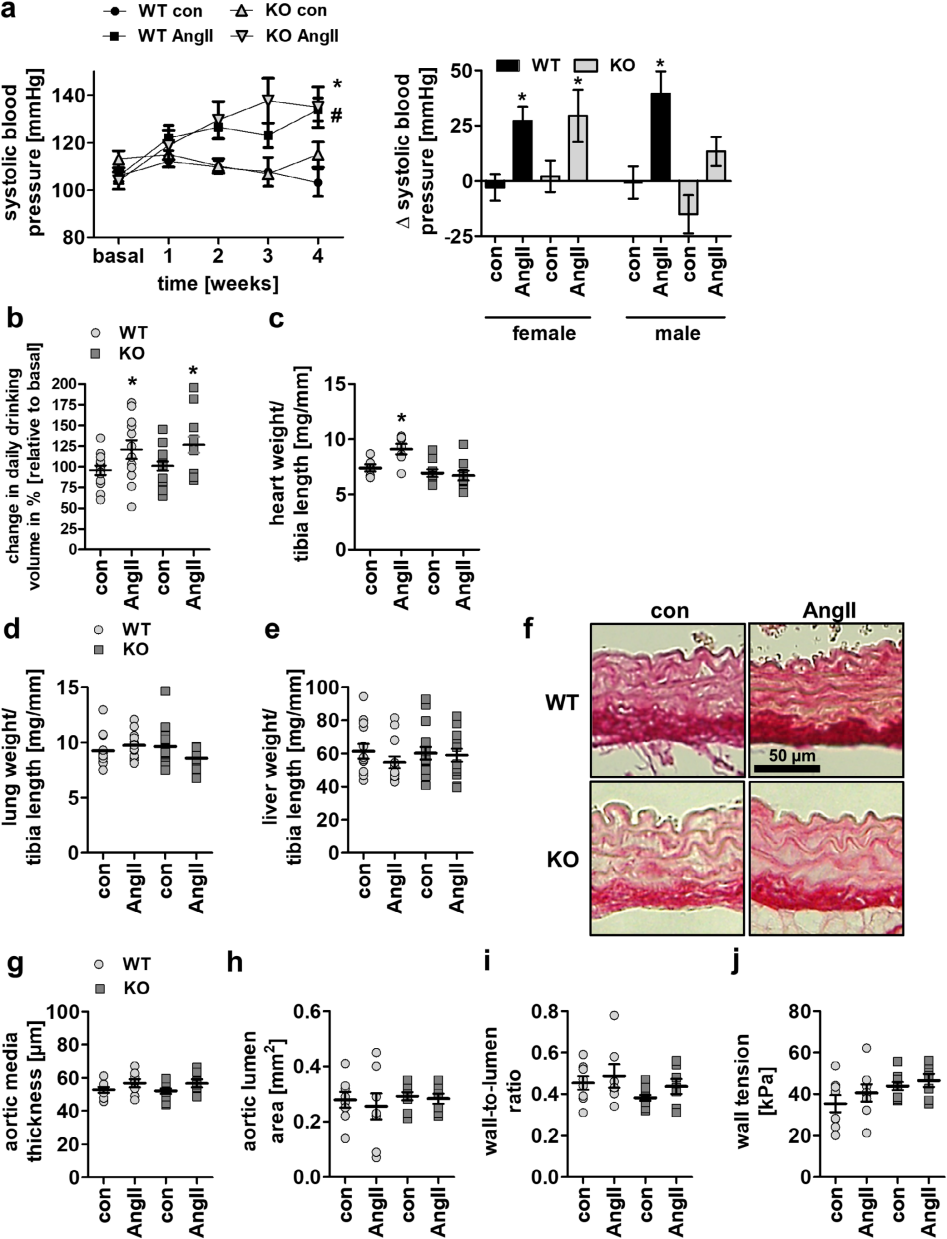
Systolic blood pressure, drinking volume, organ weight and vascular parameters of female WT and PPP3CB KO mice upon long‐term AngII treatment (a, left panel) Time course of systolic blood pressure in female mice during 4 week AngII‐ and respective control treatment. ANOVA analysis (repeated measures) was performed. *N* = 6–11 animals per conditon, *p ≤ 0.05 WT AngII to WT con; #*p* ≤ 0.05 KO AngII to KO con; repeated measures ANOVA with Dunnett's multi comparison test was used (a, right panel) Delta of systolic blood pressure after 4 weeks between female and male mice in comparison to basal values before intervention. Male data shown previously in Nolze et al. [14]. *p ≤ 0.05 AngII to respective basal value (b) Daily drinking volume of female mice under basal and treated conditions. (c–e) Summary of (c) heart weight and (d) lung weight and (e) liver weight of the mice from long‐term AngII‐treatment experiments. (f) Representative histological staining (Picro Sirius Red) of aortic sections from female WT and PPP3CB KO animals after 4 week AngII‐treatment. (g–j) Quantification of (g) aortic media thickness, (h) aortic lumen area, (i) wall‐to‐lumen ratio and (j) wall tension from histological staining of aortic rings from female mice. *N* = 6–11 animals per conditon, *p ≤ 0.05. The graphs show the mean ± SEM. One way ANOVA analysis or student's *t*‐test were performed.

To assess the role of AngII and calcineurin in the pathogenesis of hypertension in females, 4–6 months old WT female mice and corresponding female PPP3CB KO littermates were treated with 500 ng/kg/min AngII for 28 days through osmotic minipumps. We found a slow and moderate increase in systolic blood pressure (SBP) upon AngII‐treatment in WT and PPP3CB KO mice alike, with maximal values of 134.2 ± 4.8 and 135.2 ± 8.6 mmHg, respectively (Figure 1a, left panel). Without AngII, SBP did not change throughout the 4 weeks of the experiment (Figure 1a, left panel). Compared to male mice, measured in a previous publication, blood pressure increase was PPP3CB‐independent (Figure 1a, right panel) [14].

AngII led to a comparable increase in water intake in WT and KO animals (Figure 1b) but to a rise in heart weight detectable in WT but not KO animals (Figure 1c). Lung, liver, and kidney weight as well as overall body weight and tibia length were not altered in the four different mouse groups (Figure 1d,e and Figure S1a–c). AngII stimulation had no effect on aortic media thickness, aortic lumen area, wall‐to‐lumen ratio, or aortic wall tension in WT and PPP3CB KO female animals (Figure 1f–j).

### 2.2 Long‐Term AngII Treatment Does Not Affect the Acute Response to Vasoconstrictors or Vasodilators in Female Mice (But Leads to Endothelial Dysfunction in Male Mice)

Subsequently, we evaluated the response of large vessels (aortae) to vasoconstrictors and vasodilators after long‐term AngII treatment in female and male WT and PPP3CB KO mice by wire myography and in small vessels (mesenteric arteries) with pressure myography. To assure vessel viability, we determined potassium chloride‐induced contraction that showed no major differences in all experimental groups (Figure S4a–d). This maximal value was set as “100% vasoconstriction”. For vasodilation experiments, we used the thromboxane analogue U46619 for pre‐contraction. Female aortic samples showed a slightly reduced U46619‐induced contraction compared to males whereas mesenteric arteries did not (Figure S4e–f). Full reversal of the thromboxane effect by vasodilators means “100% vasodilation”.

**FIGURE 2:**
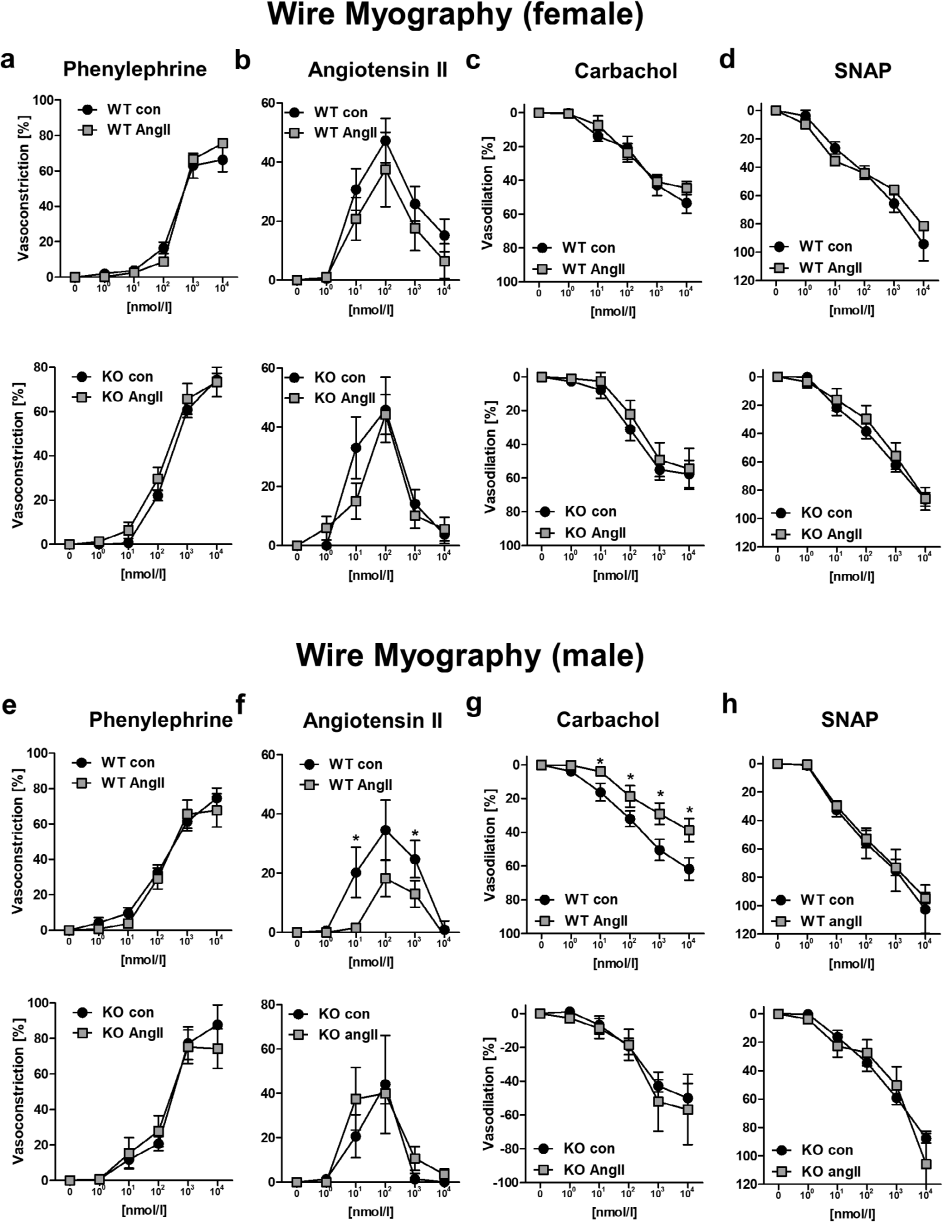
Wire myography measurements of aortic rings from female and male WT and PPP3CB KO animals treated 4 weeks with AngII or respective controls. (a) Aortic rings from female mice were treated with increasing concentrations of alpha‐adrenergic receptor agonist phenylephrine, or (b) rising concentrations of Angiotensin II. After precontraction with U46619, (c) endothelium‐dependent vasodilator carbachol or (d) endothelium‐independent vasodilator SNAP were administered. (e) Aortic rings of male mice were treated with rising concentrations of alpha‐adrenergic receptor agonist phenylephrine, (f) with rising concentrations of AngII after precontraction with U46619, (g) with endothelium‐dependent vasodilator carbachol and (h) with endothelium‐independent vasodilator SNAP. *N* = 6–8 animals per condition, *n* = 12–16 vessels per condition, *p ≤ 0.05. Force generation is depicted based on maximum contraction with potassium chloride (=100% contraction) or with a complete reversal of the U46619‐induced contraction (=100% vasodilation). The graphs show the mean ± SEM. One way ANOVA analysis was performed.

In female mice, vasoconstriction induced by acute application of phenylephrine or AngII was of comparable magnitude in all experimental groups (Figure 2a,b and Figure S2a,b). Furthermore, we found no differences in endothelium‐dependent or ‐independent vasodilation by carbachol or SNAP in WT and PPP3CB KO animals with and without long‐term AngII‐treatment (Figure 2c,d and Figure S2c,d).

In male KO and WT littermates measured simultaneously, we also found no differences in phenylephrine‐induced contraction between the different experimental groups (Figure 2e and Figure S2e). However, the response to acute AngII‐stimulation was reduced after long‐term AngII‐treatment in WT but not in PPP3CB KO mice suggesting a reduced potential to react to acute AngII‐administration after chronic AngII‐infusion (Figure 2f and Figure S2f). Additionally, WT and PPP3CB KO animals showed no difference in endothelium‐dependent vasodilation under control conditions by carbachol (Figure S2g). After long‐term AngII‐treatment, vasodilation was reduced in the aortae of WT, but not in PPP3CB KO male mice (Figure 2g). Endothelium‐independent vasodilation by SNAP was unaltered in the four groups (Figure 2h and Figure S2h).

**FIGURE 3:**
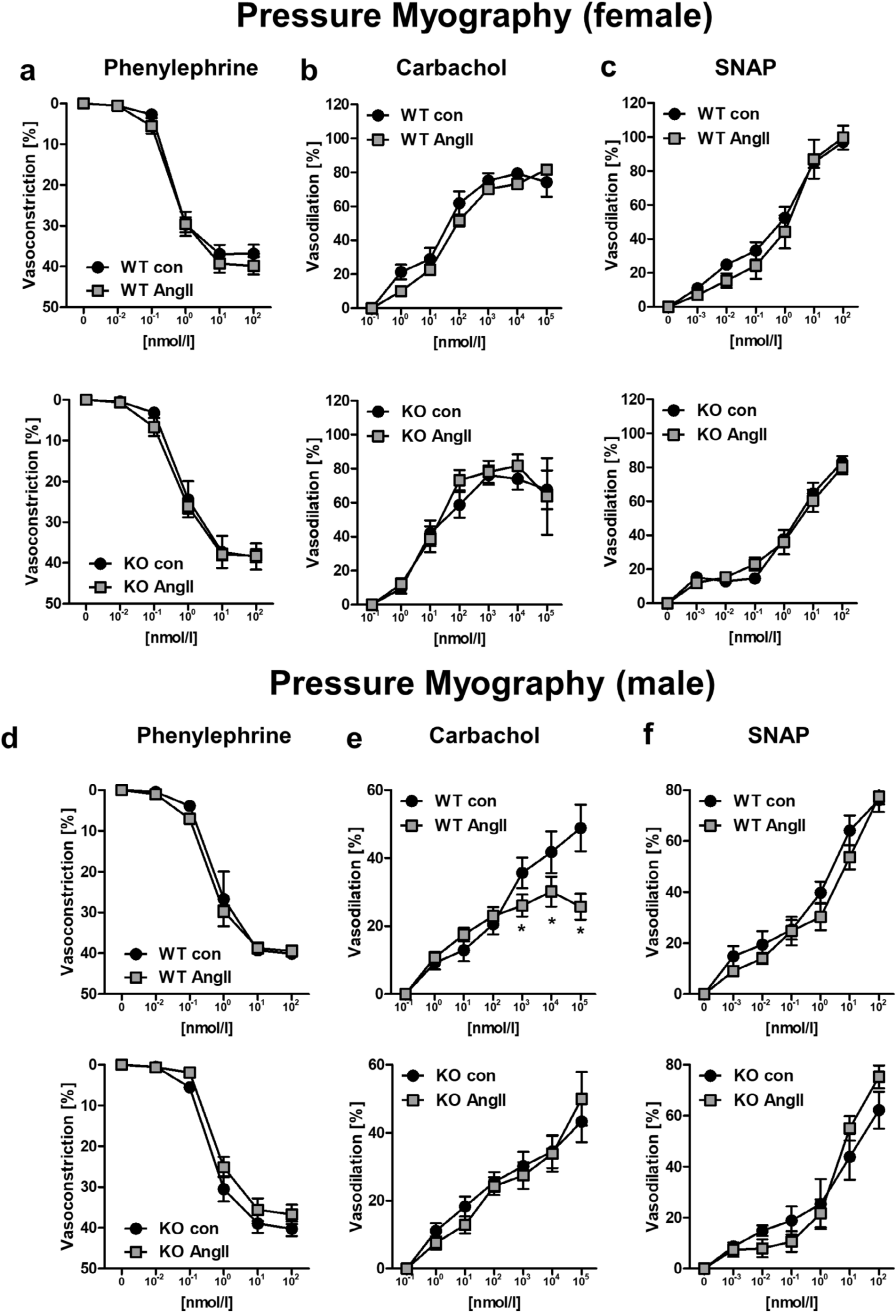
Pressure myography measurements of mesenteric arteries from female and male PPP3CB WT and KO animals upon long‐term treatment with AngII or respective controls. Mesenteric arteries from female mice were stimulated with (a) phenylephrine, (b) carbachol, and (c) SNAP while measuring vessel diameter during contraction and dilation. (d–f). Mesenteric arteries of male mice were acutely treated with (d) phenylephrine, (e) carbachol, and (f) SNAP while measuring vessel diameter during contraction and dilation. *N* = 6–8 animals per condition, *n* = 12–16 vessels per condition, *p ≤ 0.05. Vessel diameter change is depicted based on maximum contraction with potassium chloride (=100% contraction) or with a complete reversal of the U46619‐induced contraction (=100% vasodilation). The graphs show the mean ± SEM. One way ANOVA analysis was performed.

We obtained comparable results for phenylephrine, carbachol and SNAP for small mesenteric arteries in pressure myography experiments (Figure 3a–f and Figure S3a–f). Vessels from female animals exhibited no functional differences in vasoconstriction and vasodilation experiments (Figure 3a–c and Figure S3a–c) Interestingly, in male mice a reduced endothelium‐dependent vasodilation by carbachol after chronic AngII‐treatment was measured for WT but not PPP3CB KO animals (Figure 3e and Figure S3e). These results suggest that long‐term AngII‐treatment is not influencing vessel function in female mice but induces a PPP3CB‐dependent endothelial dysfunction in male mice.

### 2.3 Unlike in Male Mice, AngII Has no Effect on Wound Closure, Cell Number or Cell Diameter of aVSMCs From Female Mice

**FIGURE 4:**
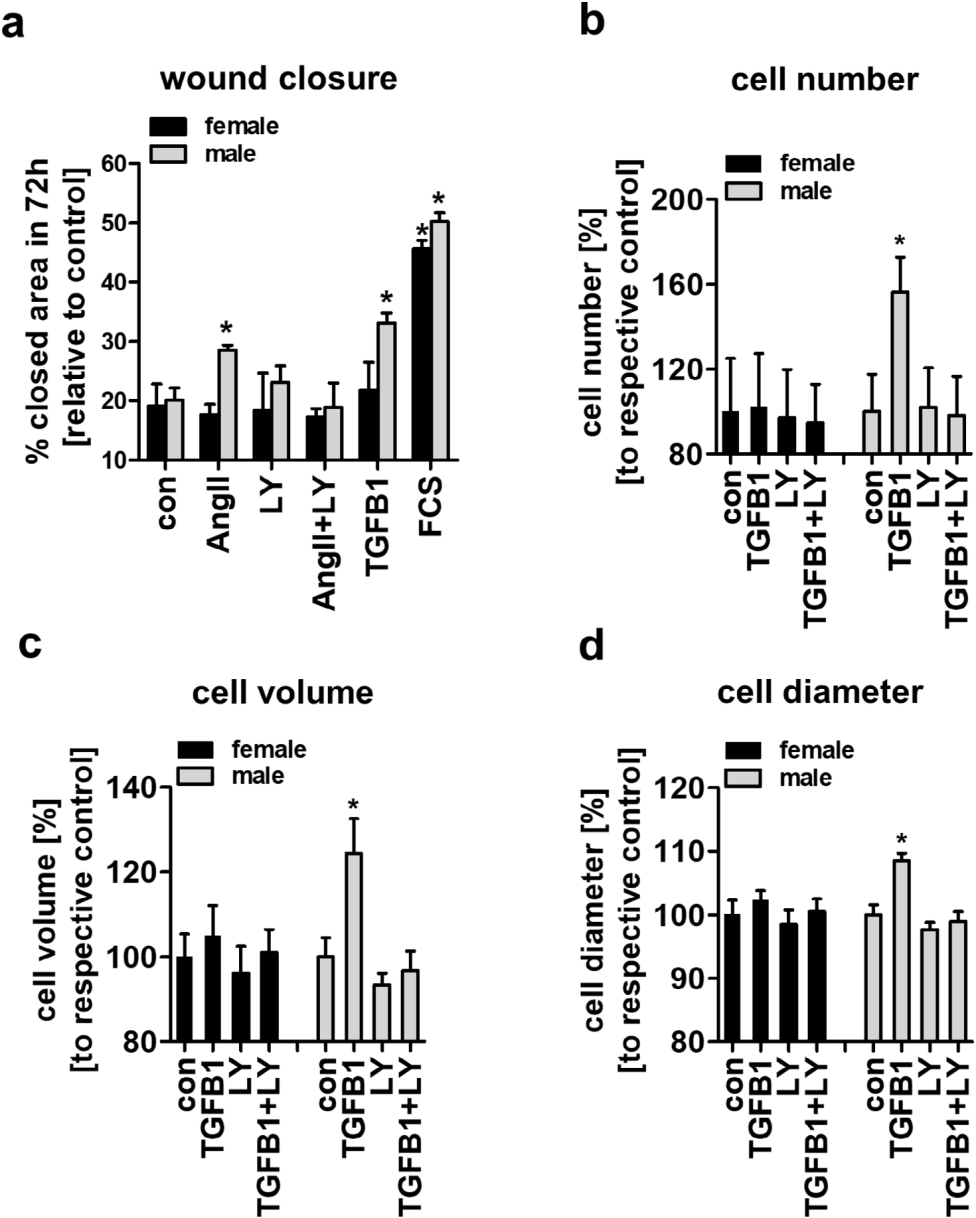
Wound closure, cell number and cell size of primary female and male murine aVSMCs after stimulation with AngII or recombinant TGFB1. (a) Wound closure of female and male WT aVSMCs was measured 72 h after applying a wound to the cell monolayer. Cells were incubated with 100 nM AngII, 3 μM TGFB1 receptor inhibitor (LY364947, LY), AngII+LY364947 (AngII+LY), 5 ng/mL recombinant TGFB1 or 2% FCS and respective controls. (b) Cell number, (c) cell volume and (d) cell diameter of female and male WT aVSMCs were measured 72 h after incubation with 5 ng/mL recombinant Tgfb1, 3 μM Tgfb1 receptor inhibitor (LY364947, LY) or recombinant TGFB1 + LY364947 (TGFB1 + LY) and respective control; *n* = 3 replicates, *p ≤ 0.05 The graphs show the mean ± SEM. One way ANOVA analysis was performed.

We next analyzed the effect of AngII on aVSMCs of female mice in a wound healing assay over 72 h (Figure 4a). Incubation with 2% fetal calf serum (FCS) and aVSMCs of male mice served as positive controls. Neither AngII nor TGFB1 had an effect on wound closure in aVSMCs of female WT mice (Figure 4a, black bars). In contrast, aVSMCs from male WT mice showed an increased wound closure after incubation with AngII or recombinant TGFB1, and the effect of AngII could be attenuated by the TGFB1 receptor inhibitor LY364947 (Figure 4a, gray bars). To further investigate and confirm the importance of TGFB1 signaling for sex differences in aVSMCs, we measured cell number, volume and diameter after TGFB1 stimulation in aVSMCs of female mice and compared the results to aVSMCs of male mice (Figure 4b–d). In female cells, TGFB1 had no effect on cell proliferation or hypertrophy, whereas male cells showed an increase in cell number, volume and diameter after TGFB1 stimulation, and this effect could be abolished by TGFB1 receptor inhibition (Figure 4b–d).

### 2.4 AngII Leads to Transcriptomal Changes in Female WT and PPP3CB KO Mice

**FIGURE 5:**
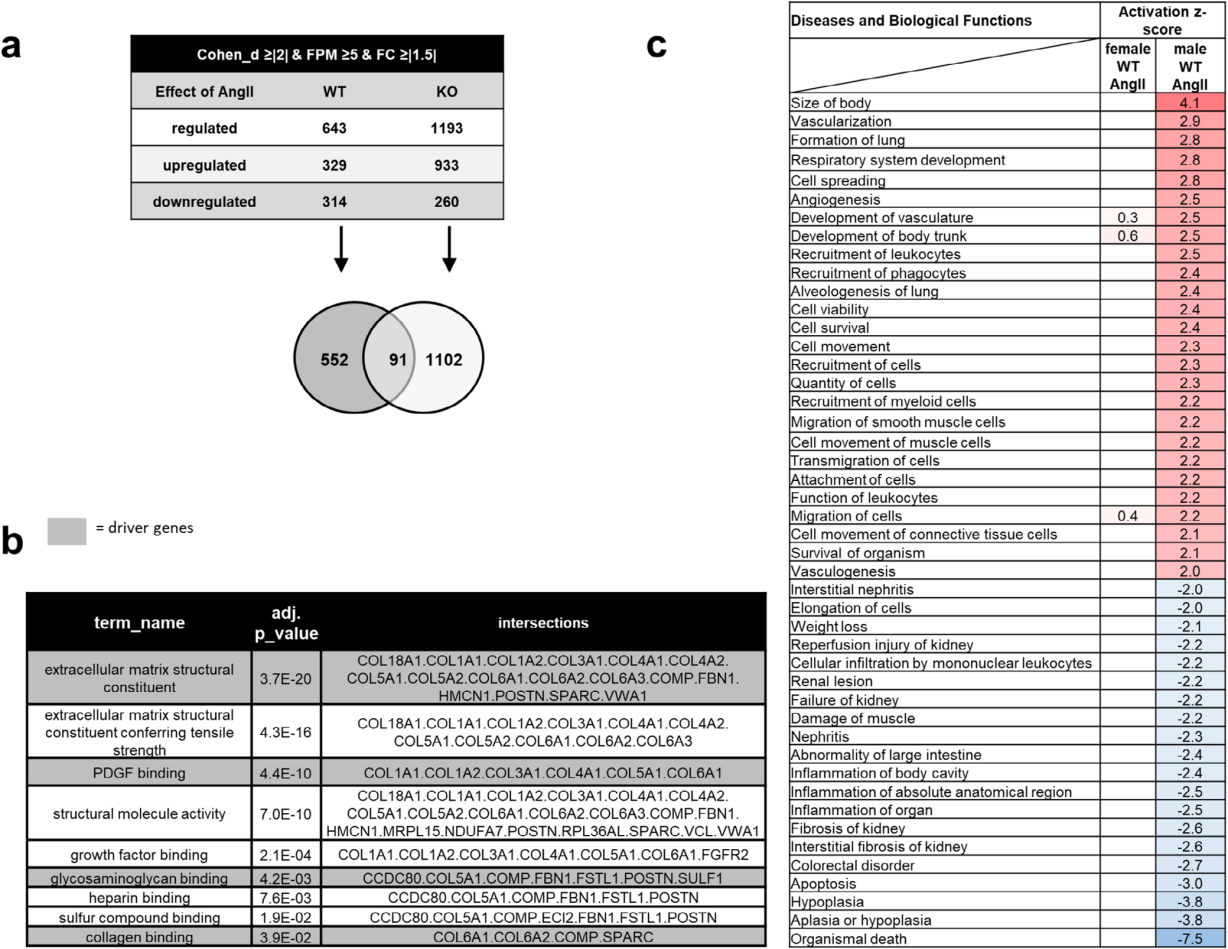
RNA‐Seq data from aorta of female WT and PPP3CB KO mice after long‐term AngII stimulation (a) RNA‐sequencing revealed AngII‐ and Calcineurin‐dependent regulation of gene expression. (b) Gene set enrichment analysis was performed with GO Profiler for the 91 AngII‐regulated genes in both genotypes. AngII‐dependently regulated genes show a high correlation to GO terms associated with extracellular matrix. (c) Overview of “diseases and biological functions” associated with AngII‐regulated genes in female and male WT animals from IPA. Results were filtered for *z*‐score ≥ |2| in the male WT AngII group. Gaps indicate terms with no z‐score value in the female WT AngII group.

**FIGURE 6:**
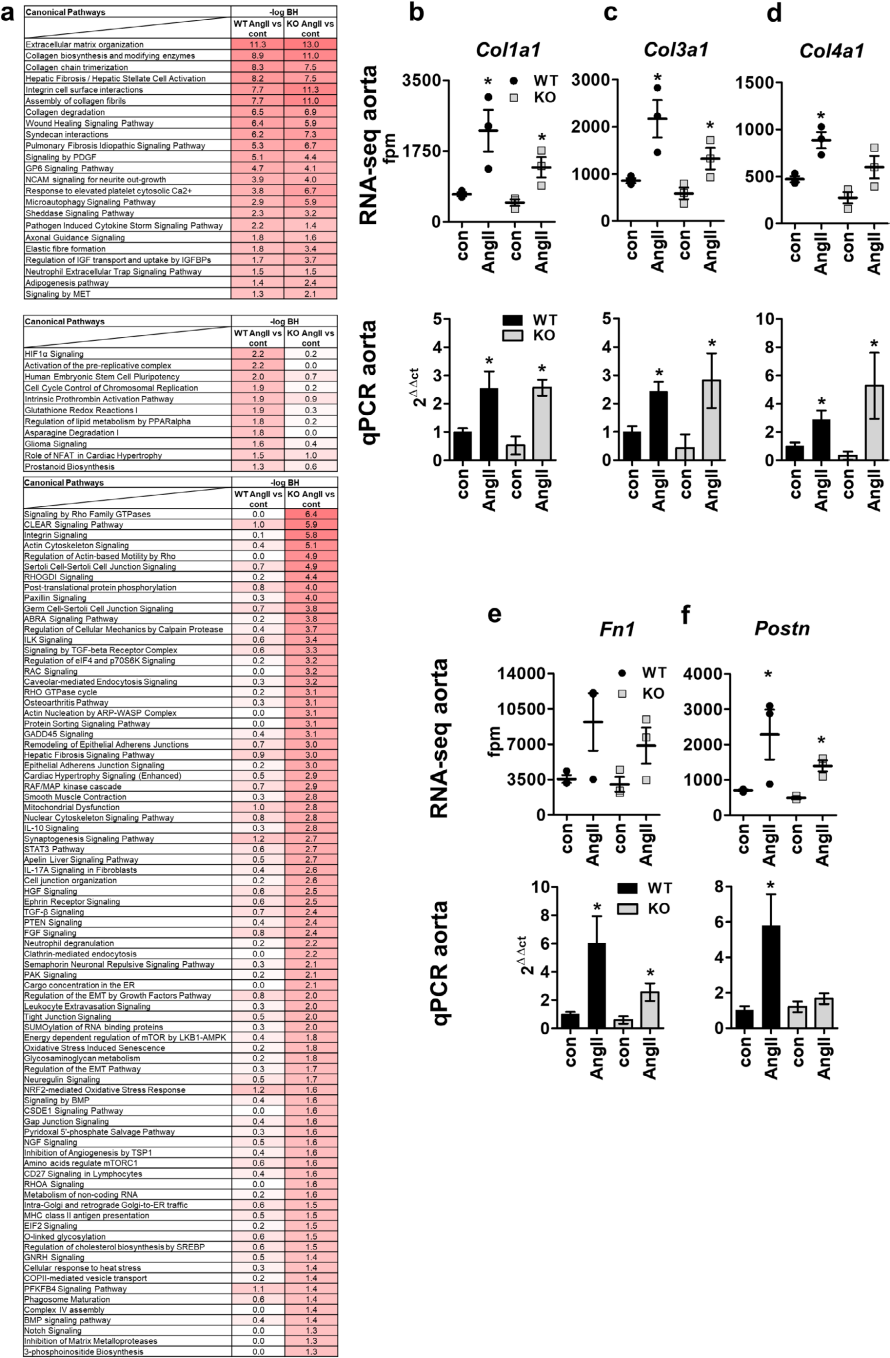
AngII‐regulated canonical pathways and genes in aorta of WT and PPP3CB KO mice in RNA‐Seq data and qPCR target gene validation. (a) Canonical pathways predicted by IPA to be regulated by AngII in female WT and KO animals (top) or just in WT animals (middle) or only in KO animals (bottom) with −log BH‐values are listed. Results were filtered for −log BH‐values ≥ 1.3 in both lists. (b–f, upper panel) RNA sequencing and qPCR validation results (b–f, lower panel) of fibrosis‐ and extracellular matrix‐associated genes *Col1a1, Col3a1, Col4a1, Fn1* and *Postn* from aortic samples of female WT and PPP3CB KO animals after long‐term AngII stimulation and respective controls are displayed. *N* = 6–11, *p ≤ 0.05. The graphs show the mean ± SEM. One way ANOVA analysis or student's *t*‐test was performed.

As no gross morphological or functional changes after AngII treatment were detectable in vessels or aVSMCs of female mice, RNA sequencing followed by Ingenuity Pathway Analysis (IPA) was performed to identify more subtle changes induced by AngII in aorta WT and PPP3CB KO mice. After filtering by a Cohen's d of ≥ |2|, a fold change ≥ ±1.5 and an abundance ≥ 5 FPM in WT control mice, 643 genes were differentially expressed in female aortic WT samples after AngII stimulation compared to control (Figure 5a), of which 329 genes were up and 314 were downregulated. Surprisingly, in PPP3CB KO animals treated with AngII, 1193 genes were differentially expressed compared to PPP3CB KO mice under control conditions (Figure 5a), with more genes up‐ than downregulated (933 vs. 260 genes). Of the AngII‐regulated genes, 91 were commonly regulated between the two genotypes (Figure 5a, Venn diagram). These commonly regulated genes show an enrichment of genes associated with GO terms like extracellular matrix structural constituents, platelet‐derived growth factor binding, glycosaminoglycan binding and collagen binding and include for example genes like collagens (Figure 5b). With IPA, we assessed the effect of AngII on diseases and biological functions in female WT animals and then compared them to the results from male mice (Figure 5c). In agreement with our experimental findings in female mice above (Figures 1, 2, 3) and based on an activated *z* scores ≥ |2| and an adjusted *p*‐values ≤ 0.05, IPA predicted only tumor‐associated terms for disease and biological functions for female mice. In male mice, on the other hand, terms like “cell viability”, “cell movement” or “migration of smooth muscle cells”, were predicted to be strongly affected by AngII (Figure 5c). For further information, canonical pathways regulated by AngII in female WT and/or KO animals were explored with IPA. Pathways dealing with extracellular matrix organization, collagen biosynthesis or integrin cell surface interactions were commonly regulated in both genotypes after AngII treatment and showed the most significant regulation. In contrast to our former results in male mice, this suggests strong but PPP3CB independent regulation. Pathways like HIF1α signaling and activation of pre‐replicative complex were only regulated by AngII in WT female animals, thus suggesting regulation by PPP3CB. AngII, on the other hand, only regulated “signaling by Rho family GTPases”, “clear signaling pathway” and “regulation of actin‐based motility by Rho” in KO animals but not in WT mice (Figure 6a). Overall, AngII signaling seems to be strongly dependent on the presence of PPP3CB. Interestingly, many of the commonly AngII‐regulated pathways in female WT and KO mice were also regulated in male WT mice but not in male KO mice (Figure S5).

We confirmed PPP3CB‐independent regulation of extracellular matrix components by AngII in females by evaluating the expression of *Col1a1, Col3a1, Col4a1, Fn1 and Postn*. In our RNA‐seq data, the expression of *Col1a1, Col3a1, Col4a1* and *Fn1* was highly increased after AngII stimulation in WT as well as PPP3CB KO animals compared to respective control (Figure 6b–e, upper panel). This regulation was confirmed by qPCR in aortic RNA samples (Figure 6b–e, lower panel). For periostin, results suggest PPP3CB‐dependent stimulation by AngII (Figure 6f).

### 2.5 Regulatory Pathways Involved in AngII Signaling Differ Between Female and Male Mice

**FIGURE 7:**
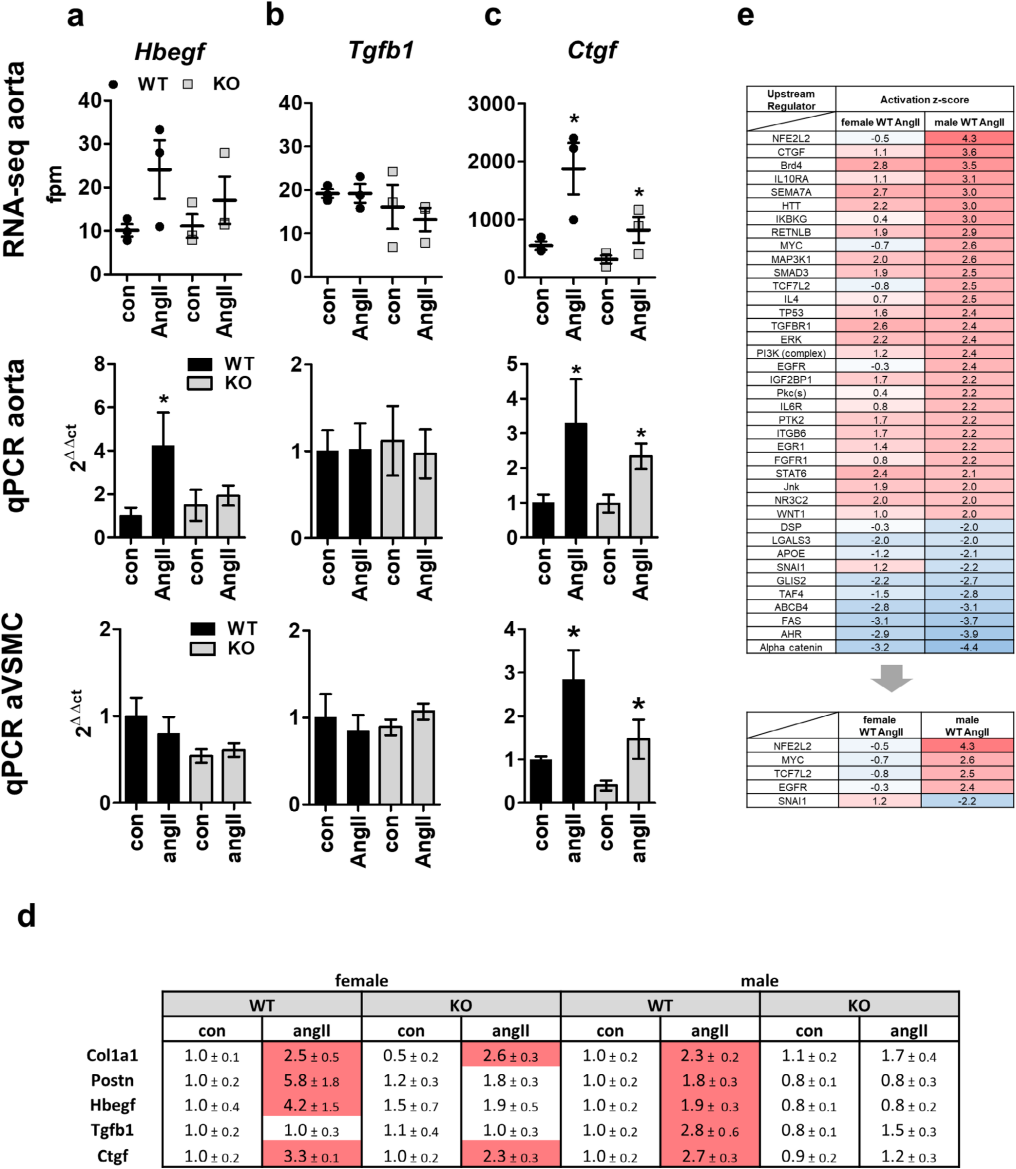
AngII‐dependent extracellular matrix regulators and overall upstream regulators. (a–c) Expression of the extracellular matrix regulators *Hbegf, Tgfb1* and *Ctgf* are shown in female samples after long‐term AngII stimulation with respective controls. (a–c, top panel) RNA sequencing results of *Hbegf, Tgfb1* and *Ctgf* from aortic samples of female WT and PPP3CB KO animals and respective controls with (a–c, middle panel) qPCR validation experiments, as well as (a–c, lower panel) results from primary aVSMCs stimulated with AngII. *N* = 3–11, *p ≤ 0.05. The graphs show the mean ± SEM. One‐way ANOVA analysis or student's *t*‐test were performed. (d) Comparison of qPCR analysis results from female and male aortic samples. Red color indicates a significant upregulation in AngII treated mice in comparison to respective control; fold change ± SEM is depicted. (e) Comparison of predicted upstream regulators of differentially expressed genes in AngII‐treated female and male WT animals with IPA. Results were filtered for *z*‐score ≥ |2| in the male WT AngII group. Upstream regulators without *z*‐score value were omitted. Positive activation *z*‐score indicates an activation of downstream genes, negative activation *z*‐score indicates an inhibitory effect on downstream targets.

To investigate common regulator pathways for extracellular matrix components as previously identified in male mice, we analyzed the expression of *Hbegf, Tgfb1*, and *Ctgf* in aortic samples by RNA sequencing (Figure 7a–c, upper panel) and validated these results by qPCR (Figure 7a–c, middle panel, primers listed in Table S1). For *Hbegf* in qPCR analysis, we found a stimulatory AngII effect in WT but not in KO mice. RNA sequencing data showed similar tendencies without reaching significance (*p* = 0.1969; Figure 7a). *Tgfb1* showed no change in expression after AngII‐stimulation compared to control in WT or PPP3CB KO animals, although basal fpm values in RNA sequencing were comparable to those of males (Figure 7b) [14]. *Ctgf* expression was enhanced in female mice upon stimulation with AngII in both genotypes (Figure 7c). As VSMC are the main effectors for regulating vascular tone, we analyzed the expression of *Hbegf, Tgfb1* and *Ctgf* in aVSMCs from female WT and KO animals. With qPCR we validated the AngII‐dependent regulation in both genotypes for *Ctgf*, and confirmed that *Tgfb1* showed no regulation at all. We found no altered expression for *Hbegf* in aVSMCs by qPCR. (Figure 7a–c, lower panel). Overall, the results from aortic samples indicate a clear sex difference in gene regulation (Figure 7d and Figure S5b).

**FIGURE 8:**
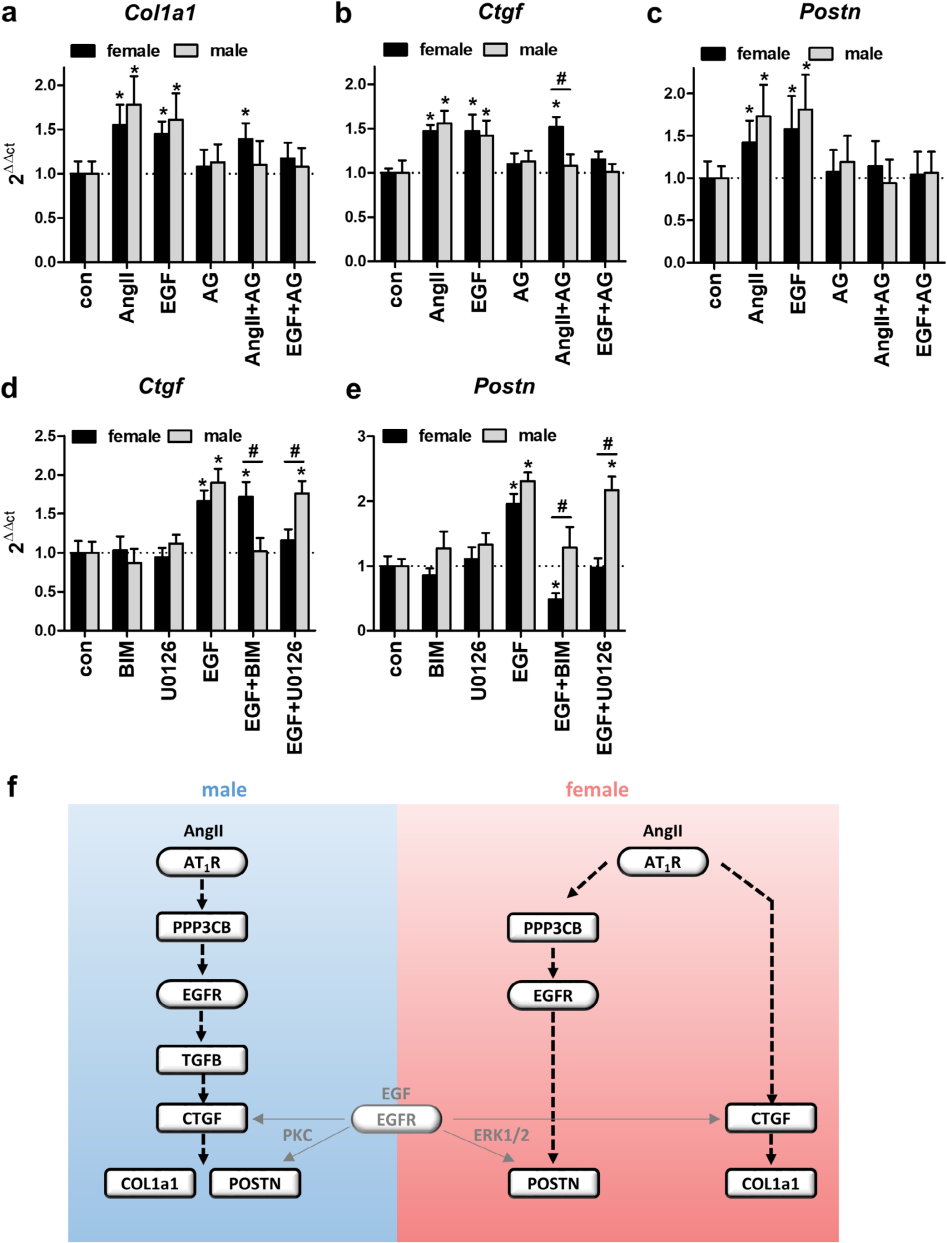
Regulation of extracellular matrix‐associated genes *Col1a1, Ctgf and Postn* in female and male aVSMCs. (a–c) Expression of *Col1a1, Ctgf* and *Postn* mRNA in aVSMCs was measured with qPCR 72 h after incubation with 100 nM AngII, 10 μg/L EGF, 1 μM AG1478 (AG), AngII+AG1478 (AngII+AG) and EGF + AG1478 (EGF + AG); *N* = 3–4, *n* = 3, *p ≤ 0.05; #*p* ≤ 0.05 indicates a significant difference between males and females. The graphs show the mean ± SEM. (d, e) Expression of *Ctgf* and *Postn* mRNA in male and female aVSMCs was measured with qPCR 72 h after incubation with 1 μM BIM‐1, 10 μM U0126, 10 μg/L EGF, EGF + BIM‐1 or EGF + U0126; *N* = 4, *n* = 4, *p ≤ 0.05; #*p* ≤ 0.05 indicates a significant difference between males and females. The graphs show the mean ± SEM. One‐way ANOVA analysis or student's *t*‐test were performed. (f) In females (red background), AngII stimulates *Col1a1* expression via *Ctgf* independently of calcineurin or EGFR. Regulation of *Postn*, on the other hand, is mediated by PPP3CB and EGFR. In males (blue background), *Col1a1* and *Postn* are both regulated by calcineurin‐EGFR‐TGFb‐CTGF signaling. Activation of EGFR by EGF was able to activate *Ctgf* and *Postn* in females and males but could be attenuated by the MEK inhibitor U0126 in females and by the PKC inhibitor BIM‐1 in males.

To find a reason for these differences in gene regulation between female and male AngII‐treated mice, we next analyzed putative upstream regulators of the differentially regulated genes for both sexes in a comparison analysis with IPA (Figure 7e). In female animals Nfe2l2 (Nrf2), MYC, TCF7L2, and EGFR signaling were predicted to be negatively regulated whereas a strong activation after AngII treatment was suggested in male animals. Snai1 (Snail1) signaling was predicted to be activated in females but downregulated in males (Figure 7e). Of the differentially expressed genes in females and males after AngII treatment, Nfe2l2 (Nrf2) and TCF7L2 (TCF/LEF), SNAI1, and CCN2 are predicted to positively regulate *Col1a1, Col3a1, Fn1*, and *Tgfb1* (Figure S6a–d). Furthermore, a putative differentially regulated upstream regulator of extracellular matrix associated genes is the epidermal growth factor receptor (EGFR). As we previously described the importance of EGFR for the regulation of extracellular matrix components in male mice, we now explored its effect on aVSMC of female mice (Figure 8).

### 2.6 Changes in Extracellular Matrix Components Are Mediated by *Ctgf* but Not by *Ppp3cb*, *Egfr*, or *Tgfb1* in aVSMCs of Female Mice

Therefore, we analyzed if the observed gene regulation of *Ctgf*, *Col1a1* and *Postn* is dependent on EGFR signaling in primary VSMCs from female and male WT animals by qPCR analysis (Figure 8a–c). *Ctgf, Col1a1*, and *Postn* mRNA expression was AngII‐ and EGF‐dependently upregulated in both sexes. Inhibiting EGFR signaling with AG1478 after AngII‐stimulation did not affect the expression in female cells but abolished the effect in male cells, suggesting that AngII‐dependent *Ctgf* and *Col1a1* expression is independent of EGFR signaling in females while being EGFR‐dependent in males (Figure 8a,b). For *Postn*, AngII‐induced mRNA expression could be abolished by AG1478 in aVSMCs of both sexes (Figure 8c). To investigate the downstream signaling of EGFR further, we stimulated aVSMCs from female and male WT animals with EGF and added inhibitors against PKC (BIM‐1) and ERK/MAPK signaling (U0126) before we determined *Ctgf* mRNA expression (Figure 8d). In female aVSMCs, blocking ERK/MAPK signaling with U0126 abolished the EGF‐mediated upregulation of *Ctgf* whereas inhibition of PKC signaling had no effect. In male aVSMCs, EGF‐dependent *Ctgf* expression was mediated by PKC but was not influenced by inhibition of ERK/MAPK signaling. These results suggest that EGFR signaling in our experimental set‐up show sex differences because *Ctgf* expression is driven by ERK/MAPK signaling in female VSMCs and by PKC signaling in male VSMCs. For *Postn* similar experiments were performed but suggest that EGF signaling in females involves ERK1/2 and possibly PKC while in males only PKC but not ERK1/2 inhibition affects *Postn* expression (Figure 8e).

## 3 Discussion

In the present study, we explore sex differences in parameters in an AngII model of hypertension. To do so we first analyze the effect of AngII on morphological and functional changes in vessels and aVSMCs of female WT and PPP3CB KO mice and compare the results to male mice. Upon AngII treatment, female mice showed only a mild, PPP3CB‐independent increase in systolic blood pressure without changes in aortic wall measurements. In contrast, their male littermates displayed a much more pronounced rise in systolic blood pressure that was accompanied by an increase in aortic media thickness and lumen area, all of which was attenuated in PPP3CB KO mice [14]. Different parameters like blood volume, heart activity, or vascular tone regulate blood pressure. AngII induced an increase in heart weight that was only detectable in WT animals but not in KO despite female animals displaying a calcineurin independent raise in blood pressure upon AngII treatment. These data suggest that the blood pressure increase is not mediated by altered heart function. While the response to vasoconstrictors and vasodilators under basal conditions was not different between males and females, long‐term AngII application induced endothelial dysfunction with reduced response to carbachol in male WT mice but not in female or in PPP3CB KO mice. Incubation of female VSMCs with AngII or TGFb1 did not affect wound closure while accelerating that of male VSMCs. Of note, female VSMCs did not respond to TGFb1 with increased cell number, volume, and diameter as male VSMCs did.

Blood pressure values were comparable to those measured by other groups administering low doses of AngII in male [17, 18, 19]. They mirror the general RAAS‐mediated sexual dimorphism in systolic blood pressure response reported in literature [20, 21, 22]. Overall, these differences between female and male mice could originate from genetic and epigenetic mechanisms as well as from differences in sex hormones and their receptors either in peripheral tissues or the central nervous system through altered sympathetic activity [23, 24, 25, 26]. Many of these parameters have a strong influence on the expression and activity of different components of the RAAS, which is known to impact the outcomes of cardiovascular diseases in males and females [16, 27, 28]. There is evidence that estrogen upregulates protective pathways like Ang‐(1–7)‐ACE2‐MasR‐AT_2_R in females and that circulating Ang‐ [1, 2, 3, 4, 5, 6, 7] levels are higher in females than in males [29]. In a study from Chao et al. the treatment of rats with 17β‐estradiol suppressed AngII‐induced cell proliferation in cardiac fibroblasts [30]. On the other hand, testosterone increases the activity of ACE and AT_1_R expression in young male mice and is a main regulator of AngII‐induced vascular dysfunction, cardiac hypertrophy and hypertension [31]. In our experiments, we did not find any significant differences in the expression levels of *At*_*1*_ *ra* or *At*_*1*_ *rb* in female or male WT and PPP3CB KO animals, whereas *At*_*2*_ *r* was not present in our data set at all (Figure S7a). But we cannot exclude changes in the activity of AT_1_R or downstream pathways. In humans, premenopausal females possess lower SBPs compared to males, which may also be caused by altered RAAS signaling with changes in vascular tone [32, 33, 34, 35]. Calcineurin previously was identified as an important mediator of AngII‐induced cardiovascular diseases, including remodeling of the vascular wall and hypertension [36, 37]. For example, in VSMCs, AngII‐induced proliferation, migration or cell senescence is regulated by calcineurin [38, 39, 40, 41, 42, 43]. Unlike for RAAS, for calcineurin the effect of sex is less clear. In some studies it was shown that the administration of estrogen in female rodents inhibited AngII‐induced cardiac hypertrophy by decreasing calcineurin activity suggesting a sex‐dependent influence of calcineurin for cardiovascular diseases [44]. In contrast, other studies describe an increase in calcineurin expression and activity in response to estrogen [45, 46].

There are studies reporting that female vessels show a reduced alpha‐adrenergic vascular tone [47] or an increased beta‐adrenergic vasodilation compared to vessels from males [48, 49]. It is also known that estrogen receptor expression in VSMCs and endothelial cells influences the functionality of the vasculature in a sex‐specific manner [50]. Endothelial dysfunction in WT male animals is caused by reduced nitric oxide (NO) production in the endothelium, which causes an increase in vascular tone that supports the development of hypertension. This could be due to a reduced activity of eNOS (Nos3) or sGC. In our RNA‐seq data, we found no difference in the expression of these genes on mRNA level between male and female mice (Figure S7b–d), but we cannot rule out altered activity due to other factors like altered calcium concentration or cGMP degradation by PDE as reported by other groups [51, 52, 53, 54].

Although in female mice AngII only led to mild hypertension as a phenotype, we found transcriptomal changes in aortic tissue, with extracellular matrix components‐associated canonical pathways showing the highest z scores in IPA. But unlike in their male littermates, expression of collagens was not attenuated in PPP3CB KO mice, indicating differences in regulation. In male mice, we reported that AngII through a calcineurin‐dependent signaling pathway leads to activation of EGFR, upregulation of *Tgfb1* and increase in *Ctgf* levels, which then lead to increased collagen expression and possibly vascular remodeling processes [14]. Ruperez et al. likewise describe Ctgf as a mediator of AngII‐induced vascular fibrosis [55]. There, administration of TGFB1 led to higher Ctgf expression in rat aVSMCs. There are controversial reports about whether Tgfb1 signaling activates Ctgf signaling or vice versa [56, 57]. In our current study in female animals, AngII increases *Ctgf* expression independently of PPP3CB and does not regulate TGFB1 expression although basal TGFb1 levels are comparable to that of males. As a possible mechanism, Ito et al. demonstrated that estrogens could disrupt Tgfb1 signaling by promoting degradation of Smad proteins [58]. We detected an increase in *Col1a1* and *Col3a1* expression levels without any changes in gross vascular wall morphology.

*Hbegf* was AngII‐ and calcineurin dependently regulated in aortae but not in VSMCs from female animals in our current study. Unlike in males, the *Hbegf*‐EGFR axis does not seem to be responsible for *Ctgf* and *collagen* upregulation but for upregulation of *Postn*. There are reports that *Hbegf* promotes *Snai1* expression [59, 60, 61]. Accordingly, in IPA, an increase in the activation of the transcription factor Snai1 was predicted as a putative upstream regulatory event in female but not in male aortic tissue (Figure 7d). Furthermore, in male WT aortic samples, the transcription factor Nrf2 was predicted to be activated after AngII stimulation, whereas it was not activated in females under the same conditions (Figure 7d). In literature there are controversial reports regarding NRF2 and EGFR signaling. On the one hand, NRF2 is known to activate EGFR signaling; on the other hand, EGFR activates NRF2 [62, 63]. Although the exact mechanism behind Nrf2 signaling in aortic samples is not completely clear, we hypothesize that upon AngII‐mediated PPP3CB activation, Nrf2 could promote EGFR signaling in male samples.

There are many reports about sex differences in EGFR expression and signaling [64, 65]. Zhang et al. found a higher EGFR expression and signaling activity in male mice in comparison to female mice [65], while in our RNA‐seq data, EGFR expression levels of the female mice slightly exceeded those of male mice (Figure S7e). In our IPA, EGFR was only predicted as a positive regulator in male but not female mice (Figure 7d). In literature it has been reported that dephosphorylation of EGFR by calcineurin promotes EGFR stability and downstream signaling [66]. EGFR mediates its downstream effects via different signaling pathways including MAP3K1/ERK, PI3K/AKT, or PKC [64, 67]. AngII‐mediated upstream regulators predicted by IPA include ERK as a positive regulator in both sexes (Figure 7d). Surprisingly, PI3K and PKC were exclusively predicted to be activated in male WT mice but not in female WT animals, suggesting that downstream signaling of EGFR after AngII‐stimulation is dependent on sex. In primary VSMCs with pharmacological inhibition of respective signaling components, we found an ERK‐dependent *Ctgf* regulation in cells from females but a PKC‐dependent effect in cells of males following EGFR activation (Figure 8d).

## 4 Conclusion

Our data suggest that in different sexes, AngII has a diverging role in smooth muscle cell and aortic vessel signaling and function. In males, calcineurin (PPP3CB) signaling is responsible for the pathological effects of AngII by inducing endothelial dysfunction, vascular remodeling with VSMC migration and extracellular matrix production and hypertension. In contrast, AngII does not mediate pathological effects in female mice and differences in coupling of AngII receptor and the calcineurin signaling cascade may be the underlying mechanism (Figure 8f). Especially, Tgfb1 signaling is calcineurin‐dependent in male mice whereas in female mice it is of minor importance. Consequently, inhibition of PPP3CB therefore may be an additional tool to target cardiovascular diseases in males while females may profit more from inhibition of PPP3CB‐independent ERK signaling.

## 5 Materials and Methods

### 5.1 Animal Experiments

PPP3CB KO mice were purchased from Jackson laboratory (JAX; B6,129S6‐Ppp3cbtm1Jmk/J; stock #009066) and kept under a 12 h/12 h light/dark regime with a room temperature of 20°C ± 1°C. According to JAX protocols, genotyping was performed. The experiments were conducted with 4–6 months old male and female mice. The study was blinded and randomized. AngII‐releasing osmotic minipumps (500 ng/kg bodyweight/min) were implanted after isoflurane anesthesia (2% in 100% O_2_, 1 L/min). After 4 weeks of AngII infusion animals were euthanized by pentobarbital injection and cervical dislocation, organs were excised, organ weights were determined and aortae as well as mesenteric arteries were used for myography measurements or histological stainings.

### 5.2 Tail Cuff Measurements

Systolic blood pressure was determined by tail cuff measurements as described previously [14].

### 5.3 Mulvany Wire Myography

The method was previously described in Stern et al. [68]. Aortic sections (3 mm) were mounted with a wire (75 μm in diameter) in a DMT wire myograph (Aarhus, Denmark). After rinsing in Tyrode's solution (119.9 mM NaCl, 5.4 mM KCl, 1.1 mM MgCl_2_, 22.6 mM NaHCO_3_, 0.42 mM NaH_2_PO_4_, 2.5 mM CaCl_2_, 5.05 mM glucose, 0.28 mM ascorbic acid, 0.05 mM EDTA) for 30 min and 10 min pretension which resulted in a force of 12 mN, aortic sections were incubated in 25 mM KCl for assessing vessel viability and contraction ability. After rinsing 5 times, which resulted in an approx. 100000‐fold dilution of the substance, the next substance was applied. Thoracic aortic sections were incubated with increasing concentrations of phenylephrine and abdominal aortic sections with AngII (1 nM–10 μM) for vasoconstriction. After rinsing 5 times with Tyrode's solution, aortic sections were treated with 30 nM U46619 for precontraction. After stable force development of the previously applied vasoconstrictor, increasing concentrations of S‐nitroso‐n‐acetyl‐penicillamine (SNAP) or carbachol (1 nM–10 μM) for vasodilation were administered.

### 5.4 Pressure Myography

The method was previously described in Stern et al. [68]. After removal of the mesenteric bed and transfer to physiological salt solution at 4°C (with 20% O_2_ and 5% CO_2_), mesenteric arteries (third or fourth order) were mounted on glass cannulas to allow perfusion at physiological pressures (inner pressure 60 mmHg, outer pressure 45 mmHg). The mesenteric arteries were superfused continuously with Krebs–Henseleit solution (20% O_2_, 5% CO_2_); pH, 7.4; 37°C made of 119 mM NaCl, 4.7 mM KCl, 25 mM NaHCO_3_, 1.2 mM KH_2_PO_4_, 1.6 mM CaCl_2_, 1.2 mM MgSO_4_, 0.03 mM EDTA and 11.1 mM glucose. By stepwise pressurizing to 20, 40, 60, 80, or 100 mmHg using the servo control system, the wake‐up procedure was carried out according to the manufacturer's instructions. Vessel diameters (basal: male 275 ± 91 μm, basal: female 277 ± 105 μm) were measured with a Zeiss Axiovert microscope (Oberkochen, Germany) and a data acquisition and analysis system (DMT, Hinnerup, Denmark). A 60 mM KCl challenge was performed after 45 to 60 min equilibration. Then dose–response curves were measured by using increasing concentrations of the vasoconstrictor phenylephrine. For vasodilation experiments, a precontraction with 30 nM U46619 was performed and from the point of stable force generation, vasodilators carbachol or SNAP were applied.

### 5.5 Histological Staining

For histological analysis of vessel morphology, aortic sections of long‐term AngII‐treated female mice and respective controls were paraformaldehyde‐fixed, embedded in paraffin, cut in 5 μm sections and stained with Picro Sirius Red. Then, wall thickness was measured at least 10 different regions of the section. Inner and outer circumference were measured for calculation of wall‐to‐lumen ratio and lumen area. Using Laplace Law (T(wall tension) = P(blood pressure) × r(radius)/d(wall thickness)) with taking pressure values from the tail cuff measurements (1 mmHg = 133.32 Pa) the wall tension was calculated.

### 5.6 aVSMC Cell Culture

Cell culture was performed as previously described [14]. In short, after isolation, aortic vascular smooth muscle cells were cultured in 1:1 DMEM/Ham's F12 supplemented with IHKE and maintained at 37°C. For stimulation experiments, cells were maintained for 24 h under serum starvation and incubated as indicated. All experiments were performed on cells that underwent up to 10 passages.

### 5.7 Wound Healing Assay and Cell Proliferation Measurements

Wound healing was assessed after 72 h with a Fiji plugin and cell proliferation was determined with the CASY Cell Counter & Analyzer (OLS, Bremen, Germany) as described previously [14].

### 5.8 Quantitative Real‐Time PCR Analysis

RNA was extracted with TRIzol (Life Technologies), reverse transcribed with SuperScriptII‐RT (Life Technologies), and then analyzed using Platinum SYBR Green qPCR Supermix UDG (Life Technologies) and primers listed in Table S1 in an Applied Biosystems 7900HT Fast Real‐Time PCR System.

### 5.9 RNA Sequencing and IPA

Sequencing of total aortic RNA from female mice was performed at the sequencing core facility of the IZKF Leipzig (Faculty of Medicine, University Leipzig, Germany). Normalization and differential expression analysis were performed using the R package EdgeR. Gene set enrichment analysis was done by g:Profiler. Ingenuity Pathway Analysis (IPA) software (Qiagen) was used for Canonical pathway analysis, Upstream Regulator Analysis, and Diseases and Biological Functions analysis on lists of genes relevant for vascular cells and significantly regulated in AngII‐treated WT male and female mice. Terms explicitly including “tumor” and “cancer” were excluded. The featured “Comparison Analysis” tool was used to compare the different results. Results were filtered for *z*‐score ≥ |2| and/or adjusted (−log Benjamini–Hochberg (BH)) *p*‐value ≥ 1.3 in the respective groups. Filtering criteria are indicated in the respective figure legends.

### 5.10 Data and Statistical Analysis

Data are presented as mean ± SEM. Number of replicates is indicated by “*n*” and specified in the respective figure legends. “N” describes number of animals. When the normality test (Shapiro–Wilk) was successful, ANOVA analysis was performed with Holm‐Sidak or Dunnett's post hoc test. Student's *t*‐test was used when two groups were compared. For data not normally distributed, the Wilcoxon matched pairs signed rank test was used. Differences were considered significant at values of *p* ≤ 0.05.

## Author Contributions

**Alexander Nolze:** methodology, investigation, writing – original draft, writing – review and editing, visualization. **Sindy Rabe:** investigation, writing – review and editing. **Stefanie Ruhs:** investigation, writing – review and editing. **Nicole Strätz:** investigation, writing – review and editing. **Katja Quarch:** investigation, writing – review and editing. **Conny Köhler:** investigation, writing – review and editing. **Claudia Grossmann:** conceptualization, writing – review and editing, writing – original draft, funding acquisition, methodology, project administration.

## Funding

This work was supported by Wilhelm Roux Programm.

## Ethics Statement

The local government (Landesverwaltungsamt Sachsen‐Anhalt, Halle, Germany, permit number 42502‐2‐1164 MLU and 42 502‐2‐1602 MLU) approved the animal experiments. All animal procedures performed conformed to the guidelines from Directive 2010/63/EU.

## Conflicts of Interest

The authors declare no conflicts of interest.

## Supporting information


**Figure S1:** Infusion of AngII has no effect on kidney weight, body weight and tibia length. (a–c) Analysis of (a) kidney weight, (b) body length and (c) tibia length after 4 weeks of AngII treatment. *N* = 6–8 animals per group.
**Figure S2:** Genotype‐dependent comparison of force generation of aortic rings from female and male PPP3CB WT and KO animals. (a–h) Analysis of force generation in aortic rings from long‐term AngII‐stimulated female and male WT and PPP3CB KO mice and respective controls. (a–d) Aortic rings from female were treated with (a) phenylephrine, (b) Angiotensin II, (c) carbachol and (d) SNAP. (e–h) Aortic rings from male mice were treated accordingly. *N* = 6–8 animals per group, *n* = 12–16 vessels per condition.
**Figure S3:** Genotype‐dependent comparison of diameter change of mesenteric arteries from female and male PPP3CB WT and KO animals. (a–f) Change in vessel diameter assessed with pressure myography in (a–c) female and (d–f) male WT and PPP3CB KO mesenteric arteries incubated acutely with (a, d) phenylephrine, (b, e) carbachol and (c, f) SNAP. *N* = 6–8 animals per group, *n* = 12–16 vessels per condition.
**Figure S4:** Potassium‐ and TXA_2_‐dependent vasoconstriction of aortic rings and mesenteric arteries from female and male animals differs slightly between WT and PPP3CB KO animals. (a–h) Analysis of basal vasoconstriction (force generation) of aortic rings and mesenteric arteries after acute administration of (a–d) potassium chloride and (e–h) U46619 (thromboxane analogue). Force generation is depicted based on maximum contraction with potassium chloride (=100% contraction) or with a complete reversal of the U46619‐induced contraction (=100% vasodilation). Vessel diameter change is depicted based on maximum contraction with potassium chloride (=100% contraction) or with a complete reversal of the U46619‐induced contraction (=100% vasodilation). *N* = 6–8 animals per group, *n* = 12–16 vessels per condition.
**Figure S5:** IPA‐based comparison of AngII‐regulated canonical pathways in female and male aortic samples from PPP3CB WT and KO mice and summary of key regulated genes in female and male mice. (a) AngII‐dependently regulated genes (in comparison to respective controls) from female and male WT and KO mice were used in IPA for comparison of canonical pathways between the different genotypes and sexes. Pathways were filtered for *z*‐scores ≥ |2| in the “female WT AngII vs control” group (first column). (b) Comparison of RNAseq results from female and male aortic samples. Red color indicates a significant upregulation in AngII treated mice in comparison to respective control; fold change ± SEM is depicted.
**Figure S6:** Extracellular matrix associated genes are calcineurin‐dependently regulated in male aortic samples by different upstream regulators. (a, b) IPA‐based visualization of putative downstream targets of (a) NFE2L2 (NRF2), (b) TCF/LEF (c) SNAI1 and (d) CCN2 (CTGF). Figures were exported from IPA (2000–2025 QIAGEN).
**Figure S7:** Expression of *AT*_*1*_ *Ra*, At1Rb, *soluble guanylate cyclase*, *eNos* and *Egfr* shows no major differences between female and male WT and PPP3CB KO animals. (a) Expression analysis of *AT*_*1*_ *Ra* and *AT1Rb* with RNA‐seq in male and female WT and PPP3CB KO mice (*N* = 2–3 animals per group). (b–e) Expression analysis of indicated genes by RNA‐seq in aortic samples from female and male WT and PPP3CB KO animals after long‐term AngII‐treatment or respective controls, (b) *Gucy1a3*, (c) *Gucy1b3*, (d) *Nos3*, (e) *Egfr (Erbb1). N* = 3 animals per group.

**Table S1:** List of primer pairs used for qPCR experiments.

## Data Availability

The data that support the findings of this study are available from the corresponding author upon reasonable request.
