# Supplementary material for: Sex‐Dependent Effects of Angiotensin II and Calcineurin in the Vasculature of Mice
Source: Acta Physiol (Oxf). 2026 Mar 27;242(5):e70213. doi: 10.1111/apha.70213 (PMC13030993; doi:10.1111/apha.70213)
Supplement: Supplementary file 1 — Figure S1: Infusion of AngII has no effect on kidney weight, body weight and tibia length. (a–c) Analysis of (a) kidney weight, (b) body length and (c) tibia length after 4 weeks of AngII treatment. N = 6–8 animals per group. Figure S2: Genotype‐dependent comparison of force generation of aortic rings from female and male PPP3CB WT and KO animals. (a–h) Analysis of force generation in aortic rings from long‐term AngII‐stimulated female and male WT and PPP3CB KO mice and respective controls. (a–d) Aortic rings from female were treated with (a) phenylephrine, (b) Angiotensin II, (c) carbachol and (d) SNAP. (e–h) Aortic rings from male mice were treated accordingly. N = 6–8 animals per group, n = 12–16 vessels per condition. Figure S3: Genotype‐dependent comparison of diameter change of mesenteric arteries from female and male PPP3CB WT and KO animals. (a–f) Change in vessel diameter assessed with pressure myography in (a–c) female and (d–f) male WT and PPP3CB KO mesenteric arteries incubated acutely with (a, d) phenylephrine, (b, e) carbachol and (c, f) SNAP. N = 6–8 animals per group, n = 12–16 vessels per condition. Figure S4: Potassium‐ and TXA2‐dependent vasoconstriction of aortic rings and mesenteric arteries from female and male animals differs slightly between WT and PPP3CB KO animals. (a–h) Analysis of basal vasoconstriction (force generation) of aortic rings and mesenteric arteries after acute administration of (a–d) potassium chloride and (e–h) U46619 (thromboxane analogue). Force generation is depicted based on maximum contraction with potassium chloride (=100% contraction) or with a complete reversal of the U46619‐induced contraction (=100% vasodilation). Vessel diameter change is depicted based on maximum contraction with potassium chloride (=100% contraction) or with a complete reversal of the U46619‐induced contraction (=100% vasodilation). N = 6–8 animals per group, n = 12–16 vessels per condition. Figure S5: IPA‐based comparison of AngII‐regulated can [file APHA-242-e70213-s002.pdf]

Suppl. Fig. 1

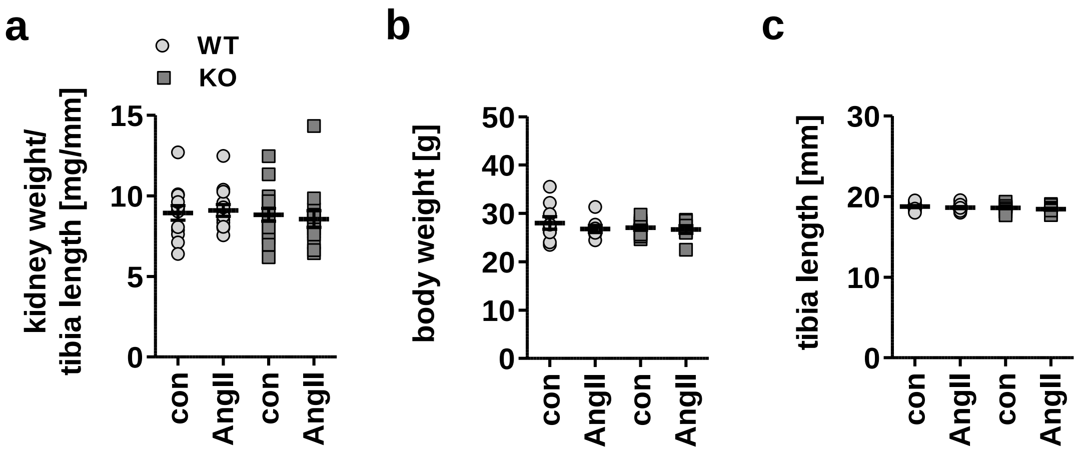

a
b
c
WT
KO
kidney weight/
tibia length [mg/mm]
body weight [g]
tibia length [mm]
15
10
5
0
50
40
30
20
10
0
30
20
10
0
con
AngII
con
AngII

Suppl. Fig. 2

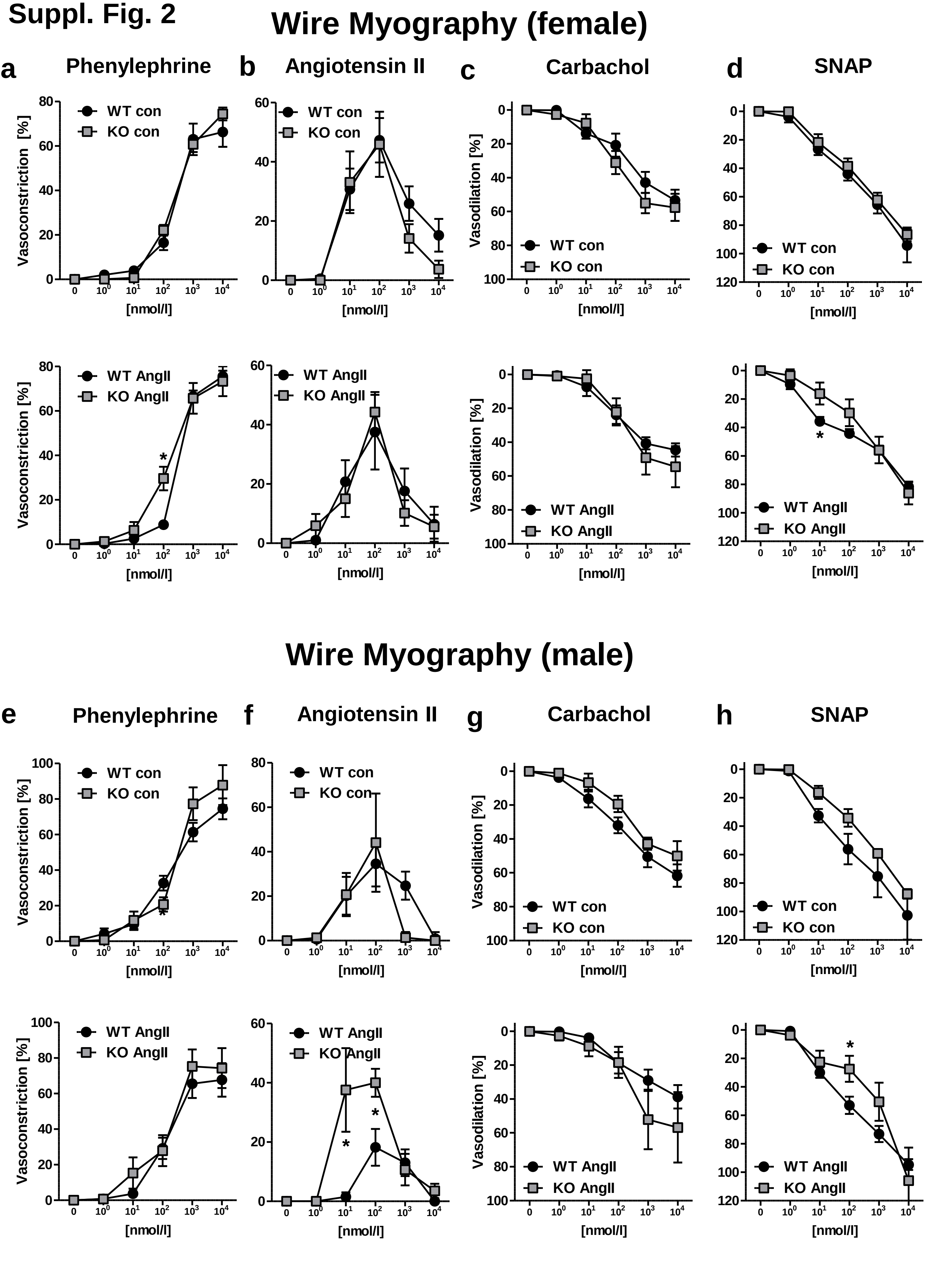

Wire Myography (female)
a
Phenylephrine
b
Angiotensin II
c
Carbachol
d
SNAP
WT con
KO con
WT AngII
KO AngII
Vasoconstriction [%]
Vasodilation [%]
[nmol/l]
*
Wire Myography (male)
e
Phenylephrine
f
Angiotensin II
g
Carbachol
h
SNAP
WT con
KO con
WT AngII
KO AngII
Vasoconstriction [%]
Vasodilation [%]
[nmol/l]
*

Suppl. Fig. 3

Pressure Myography (female)

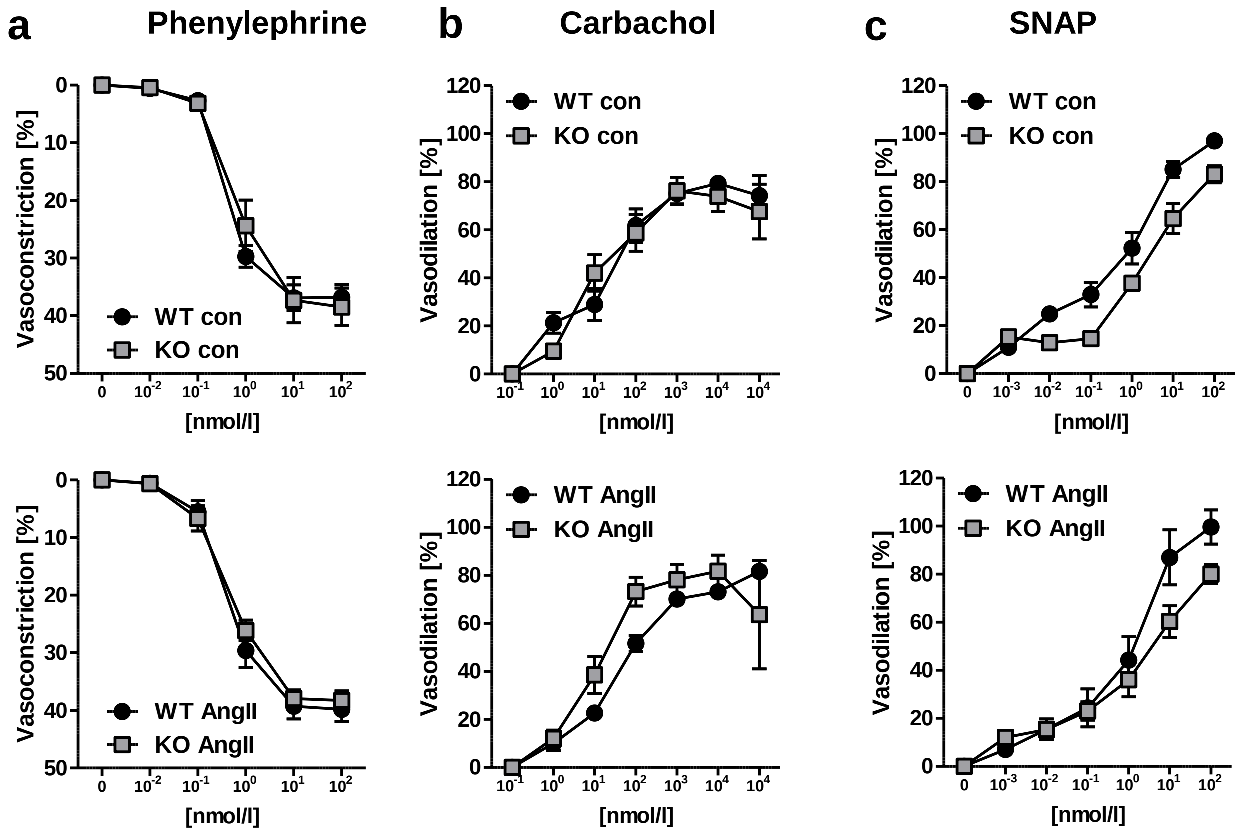

Pressure Myography (male)

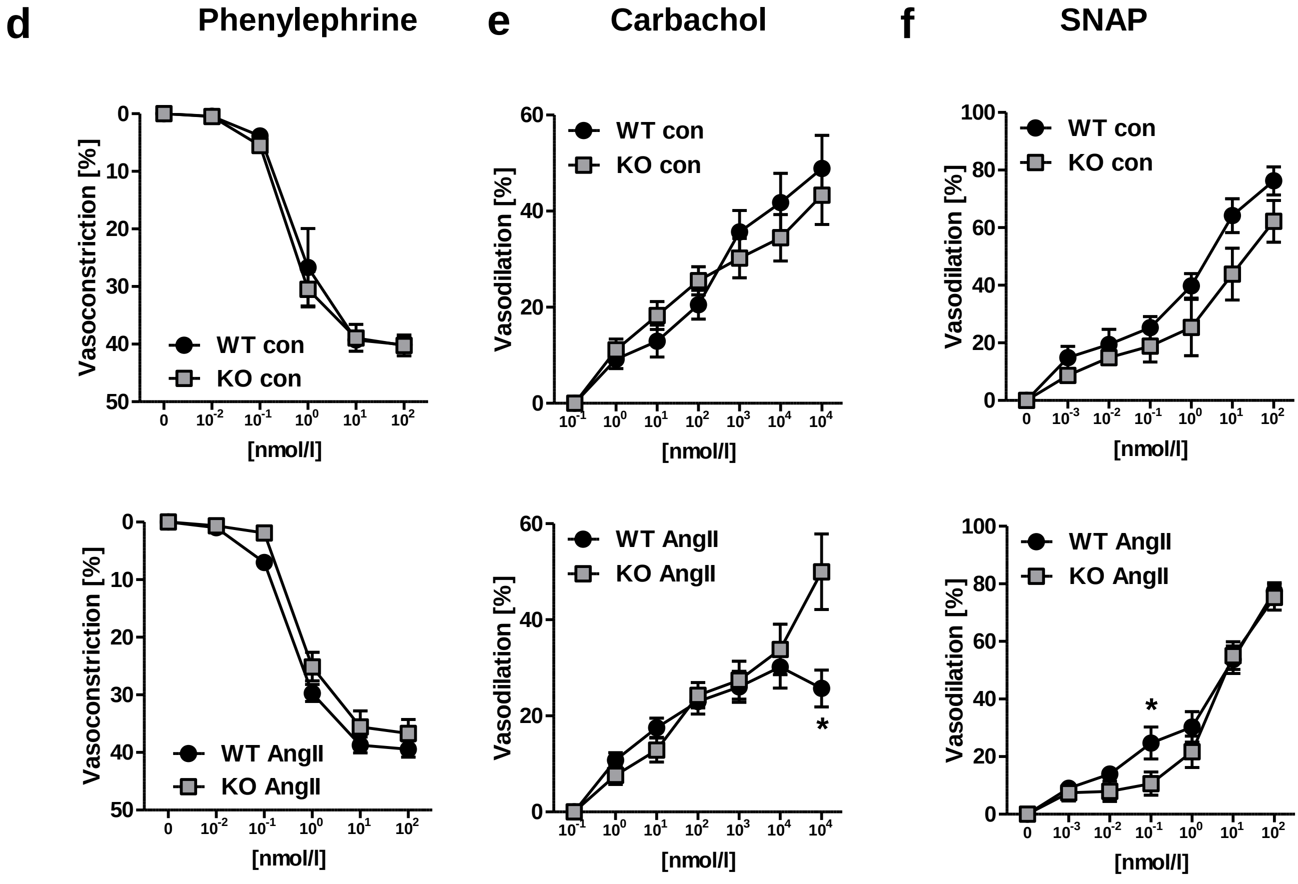

Suppl. Fig. 4

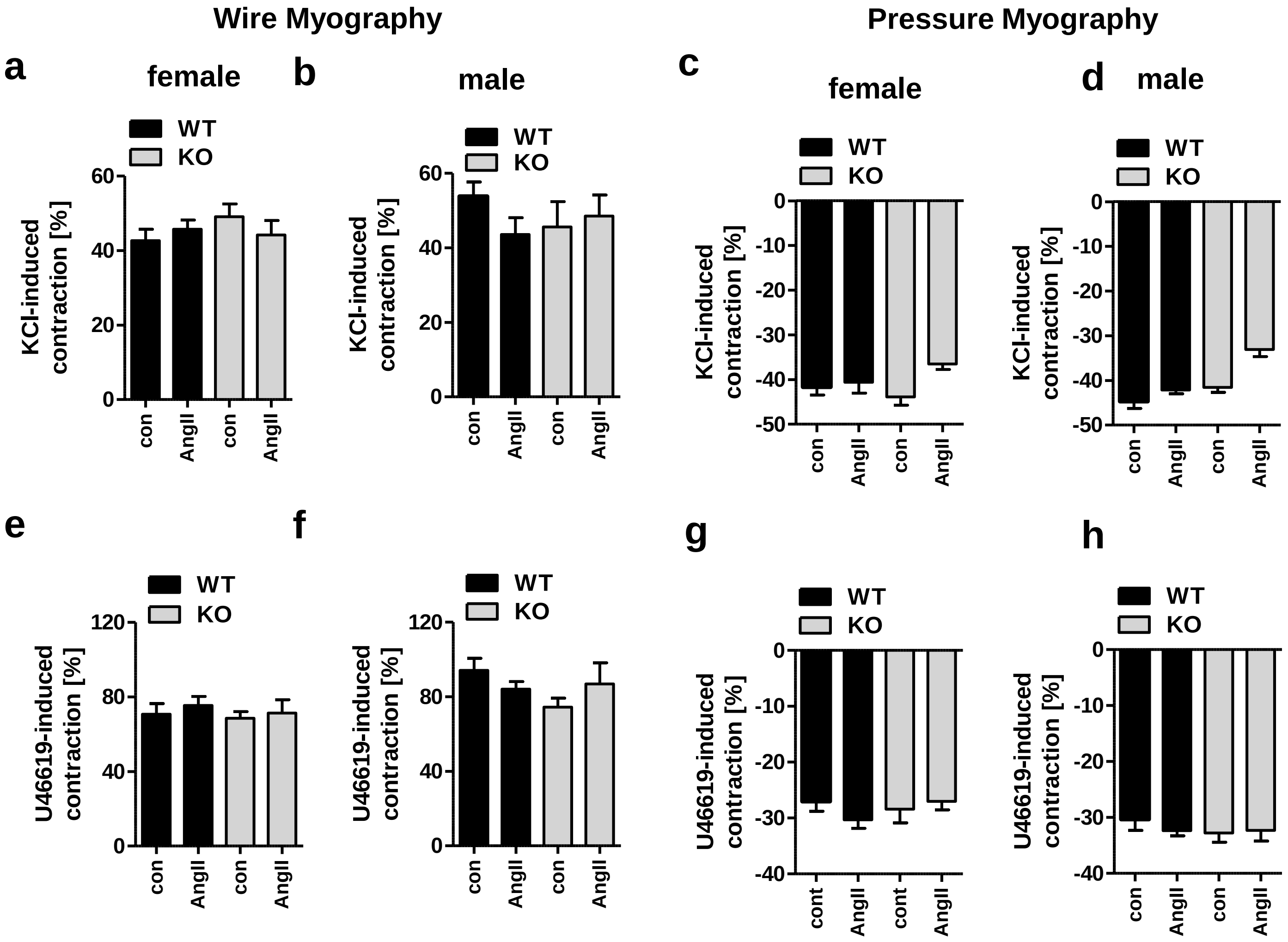

Wire Myography
Pressure Myography
a
female
WT
KO
KCl-induced contraction [%]
0
20
40
60
con
AngII
con
AngII
b
male
WT
KO
KCl-induced contraction [%]
0
20
40
60
con
AngII
con
AngII
c
female
WT
KO
KCl-induced contraction [%]
0
-10
-20
-30
-40
-50
con
AngII
con
AngII
d
male
WT
KO
KCl-induced contraction [%]
0
-10
-20
-30
-40
-50
con
AngII
con
AngII
e
WT
KO
U46619-induced contraction [%]
0
40
80
120
con
AngII
con
AngII
f
WT
KO
U46619-induced contraction [%]
0
40
80
120
con
AngII
con
AngII
g
WT
KO
U46619-induced contraction [%]
0
-10
-20
-30
-40
cont
AngII
cont
AngII
h
WT
KO
U46619-induced contraction [%]
0
-10
-20
-30
-40
con
AngII
con
AngII

Suppl. Fig. 5

a

Canonical Pathways	female: WT AngII vs. control	female: KO AngII vs. control	male: WT AngII vs. control	male: KO AngII vs. control
Pulmonary Fibrosis Idiopathic Signaling Pathway	4.60	4.96	4.75	-2.31
Wound Healing Signaling Pathway	4.38	4.60	4.64	-1.13
Role of Osteoclasts in Rheumatoid Arthritis Signaling Pathway	4.03	3.89	3.65	-1.41
Pathogen Induced Cytokine Storm Signaling Pathway	3.90	4.20	4.38	-0.38
Hepatic Fibrosis Signaling Pathway	2.83	4.16	3.31	-2.12
IL-4 Signaling	3.74	3.96	3.58	-0.45
Human Embryonic Stem Cell Pluripotency	2.67	3.74	2.67	-1.34
Nuclear Cytoskeleton Signaling Pathway	2.33	3.13	2.99	-1.41
Sertoli Cell-Sertoli Cell Junction Signaling	2.53	4.02	1.60	-1.67
Activin Inhibin Signaling Pathway	2.71	3.15	2.07	-1.00
IL-6 Signaling	2.24	3.32	0.82	-2.45
Integrin Signaling	2.24	4.04	2.00	-0.45
MHC class II antigen presentation	2.45	1.94	2.89	-1.34
ID1 Signaling Pathway	2.31	2.18	1.96	-1.13
COPI-mediated anterograde transport	2.12	2.12	1.27	-2.00
Hepatitis B Chronic Liver Pathogenesis Signaling Pathway	2.12	2.50	1.51	-1.34
MicroRNA Biogenesis Signaling Pathway	2.12	2.84	2.00	-0.45
IL-17 Signaling	2.00	1.16	1.27	0.00
PPAR Signaling	-2.00	-1.89	-1.34	2.00
Ion channel transport	-2.33	2.11	1.63	-0.82
RAR Activation	-2.32	1.00	1.23	-1.67
Neutrophil Extracellular Trap Signaling Pathway	-3.58	-0.56	-1.80	0.00

b

	female				male			
	WT		KO		WT		KO	
	con	angII	con	angII	con	angII	con	angII
**Col1a1**	681 ± 39	2255 ± 421	475 ± 64	1350 ± 205	618 ± 81	1422 ± 110	974 ± 137	1085 ± 99
**Postn**	706 ± 21	2286 ± 575	488 ± 25	1397 ± 127	575 ± 71	1314 ± 97	552 ± 63	806 ± 18
**Hbegf**	10 ± 1	24 ± 5	11 ± 2	17 ± 4	7 ± 2	23 ± 1	8 ± 1	11 ± 3
**Tgfb1**	19 ± 1	19 ± 2	16 ± 4	13 ± 2	16 ± 2	28 ± 2	21 ± 1	27 ± 2
**Ctgf**	546 ± 61	1877 ± 362	311 ± 57	817 ± 182	670 ± 56	1309 ± 92	523 ± 58	602 ± 119

Suppl. Fig. 6

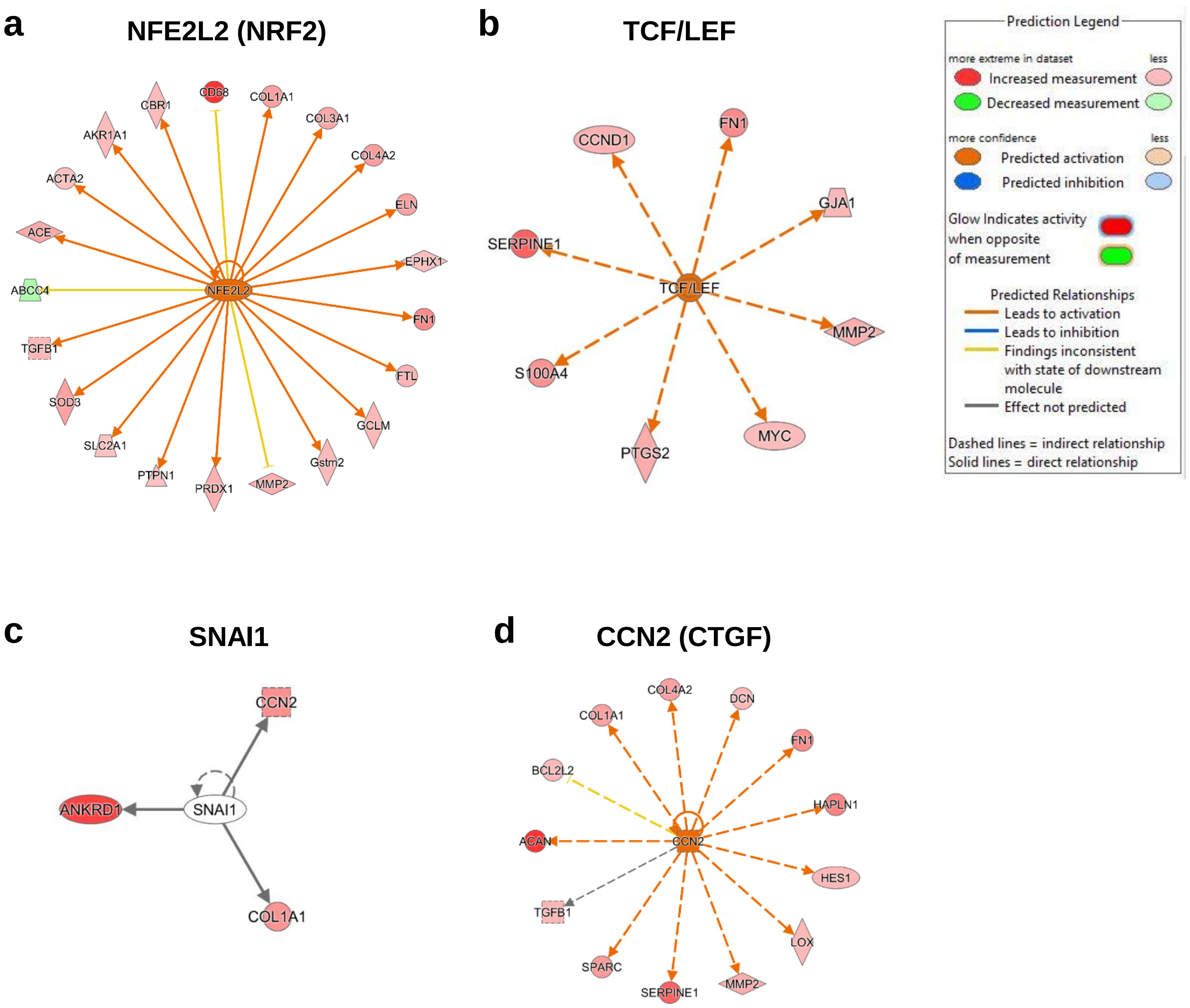
a
NFE2L2 (NRF2)
CD68
CBR1
COL1A1
COL3A1
AKR1A1
COL4A2
ACTA2
ELN
ACE
EPHX1
ABCC4
NFE2L2
FN1
TGFB1
FTL
SOD3
GCLM
SLC2A1
Gstm2
PTPN1
PRDX1
MMP2
b
TCF/LEF
CCND1
FN1
GJA1
SERPINE1
TCF/LEF
MMP2
S100A4
MYC
PTGS2
Prediction Legend
more extreme in dataset
less
Increased measurement
Decreased measurement
more confidence
less
Predicted activation
Predicted inhibition
Glow Indicates activity
when opposite
of measurement
Predicted Relationships
Leads to activation
Leads to inhibition
Findings inconsistent
with state of downstream
molecule
Effect not predicted
Dashed lines = indirect relationship
Solid lines = direct relationship
c
SNAI1
CCN2
ANKRD1
SNAI1
COL1A1
d
CCN2 (CTGF)
COL4A2
DCN
COL1A1
FN1
BCL2L2
HAPLN1
ACAN
CCN2
HES1
TGFB1
LOX
SPARC
SERPINE1
MMP2

Suppl. Fig. 7

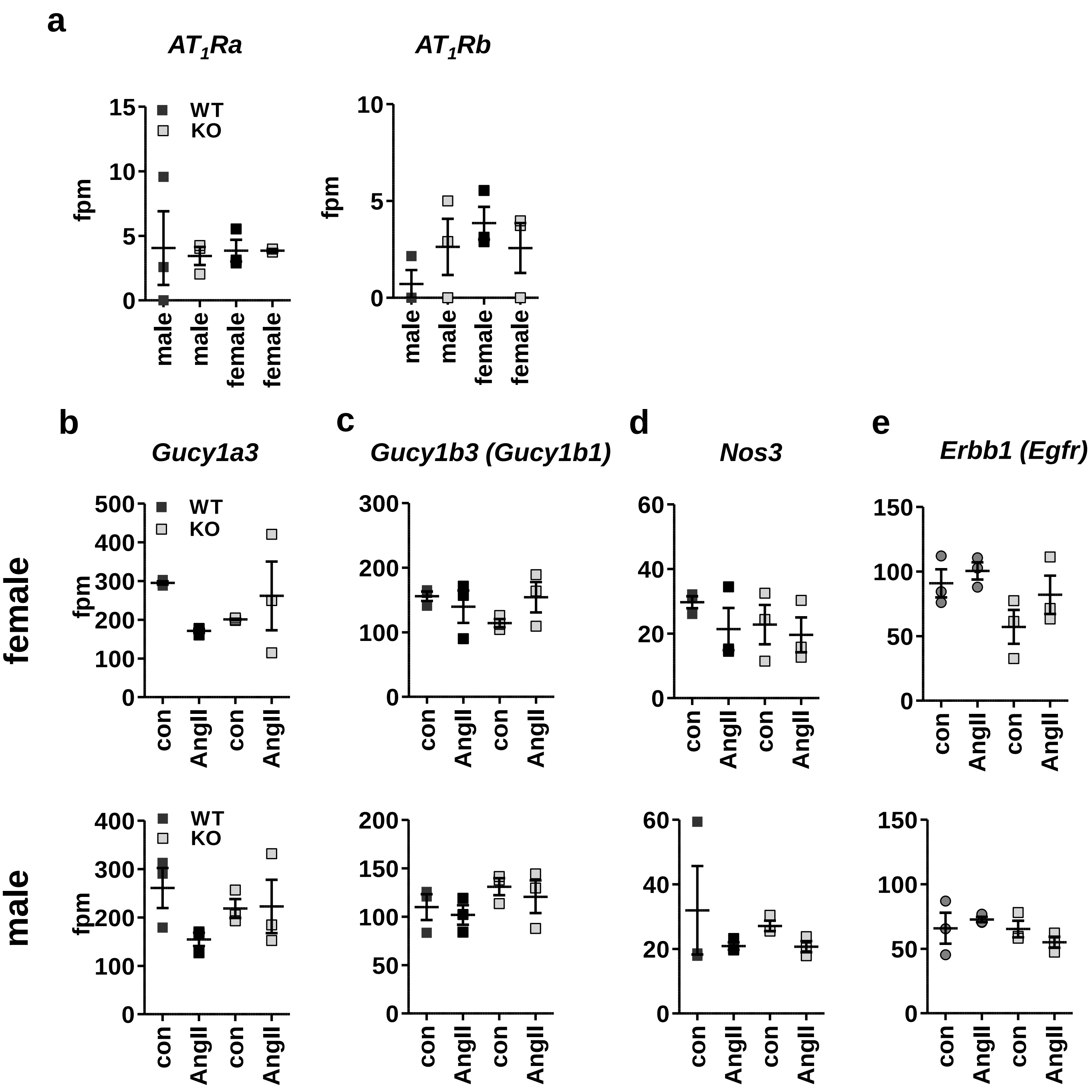
a
AT_1Ra
AT_1Rb
WT
KO
fpm
male
male
female
female
b
c
d
e
Gucy1a3
Gucy1b3 (Gucy1b1)
Nos3
Erbb1 (Egfr)
female
male
con
AngII
con
AngII
