# Supplementary material for: Sex‐Dependent Effects of Angiotensin II and Calcineurin in the Vasculature of Mice
Source: Acta Physiol (Oxf). 2026 Mar 27;242(5):e70213. doi: 10.1111/apha.70213 (PMC13030993; doi:10.1111/apha.70213)
Supplement: Supplementary file 2 — Table S1: List of primer pairs used for qPCR experiments. [file APHA-242-e70213-s001.docx]

**SUPPORTING INFORMATION**

**SUPPLEMENTAL FIGURE LEGENDS**

**Suppl. Fig. 1**

**Infusion of AngII has no effect on kidney weight, body weight and tibia length. (a– c)** Analysis of **(a)** kidney weight, **(b)** body length and (**c)** tibia length after 4 weeks of AngII treatment. N = 6 – 8 animals per group.

**Suppl. Fig. 2**

**Genotype-dependent comparison of force generation of aortic rings from female and male PPP3CB WT and KO animals.**

**(a – h)** Analysis of force generation in aortic rings from long-term AngII-stimulated female and male WT and PPP3CB KO mice and respective controls. **(a – d)** Aortic rings from female were treated with **(a)** phenylephrine, **(b)** Angiotensin II, **(c)** carbachol and **(d)** SNAP. **(e – h)** Aortic rings from male mice were treated accordingly. N = 6 – 8 animals per group, n = 12 – 16 vessels per condition.

**Suppl. Fig. 3**

**Genotype-dependent comparison of diameter change of mesenteric arteries from female and male PPP3CB WT and KO animals.**

**(a – f)** Change in vessel diameter assessed with pressure myography in **(a – c)** female and **(d – f)** male WT and PPP3CB KO mesenteric arteries incubated acutely with **(a, d)** phenylephrine, **(b, e)** carbachol and **(c, f)** SNAP. N = 6 – 8 animals per group, n = 12 – 16 vessels per condition.

**Suppl. Fig. 4**

**Potassium- and TXA_2_-dependent vasoconstriction of aortic rings and mesenteric arteries from female and male animals differs slightly between WT and PPP3CB KO animals.**

**(a – h)** Analysis of basal vasoconstriction (force generation) of aortic rings and mesenteric arteries after acute administration of **(a – d)** potassium chloride and **(e – h)** U46619 (thromboxane analogue). Force generation is depicted based on maximum contraction with potassium chloride (= 100% contraction) or with a complete reversal of the U46619-induced contraction (= 100% vasodilation). Vessel diameter change is depicted based on maximum contraction with potassium chloride (= 100% contraction) or with a complete reversal of the U46619-induced contraction (= 100% vasodilation). N = 6 – 8 animals per group, n = 12 – 16 vessels per condition.

**Suppl. Fig. 5**

**IPA-based comparison of AngII-regulated canonical pathways in female and male aortic samples from PPP3CB WT and KO mice and summary of key regulated genes in female and male mice.**

(a) AngII-dependently regulated genes (in comparison to respective controls) from female and male WT and KO mice were used in IPA for comparison of canonical pathways between the different genotypes and sexes. Pathways were filtered for z-scores ≥ |2| in the “female WT AngII vs control” group (first column). (b) Comparison of RNAseq results from female and male aortic samples. Red color indicates a significant upregulation in AngII treated mice in comparison to respective control; fold change±SEM is depicted.

**Suppl. Fig. 6**

**Extracellular matrix associated genes are calcineurin-dependently regulated in male aortic samples by different upstream regulators.**

**(a, b)** IPA-based visualization of putative downstream targets of **(a)** NFE2L2 (NRF2), **(b)** TCF/LEF **(c)** SNAI1 and **(d)** CCN2 (CTGF). Figures were exported from IPA (© 2000 -2025 QIAGEN).

**Suppl. Fig. 7**

**Expression of *AT_1_Ra*, At1Rb, *soluble guanylate cyclase*, *eNos* and *Egfr* shows no major differences between female and male WT and PPP3CB KO animals.**

**(a)** Expression analysis of *AT_1_Ra* and *AT1Rb* with RNA-seq in male and female WT and PPP3CB KO mice (N = 2 – 3 animals per group). **(b – e)** Expression analysis of indicated genes by RNA-seq in aortic samples from female and male WT and PPP3CB KO animals after long-term AngII-treatment or respective controls, **(b)** *Gucy1a3*, **(c)** *Gucy1b3*, **(d)** *Nos3,* **(e)** *Egfr (Erbb1).* N = 3 animals per group.

**Suppl. Table 1**

**List of primer pairs used for qPCR experiments.**

| Primer Name | Forward Sequence | Reverse Sequence |
| --- | --- | --- |
|  |  |  |
| *Eef2* | TTCTGATGATGGGCCGCTAC | AAGGTAGTGATGGTCCCCGT |
| *Ctgf* | GCTTGGCGATTTTAGGTGTC | CAGACTGGAGAAGCAGAGCC |
| *Tgfb1* | TTTGGAGCCTGGACACACAG | TGGACAACTGCTCCACCTTG |
| *Hbegf* | GACCCATGCCTCAGGAAATA | TGAGAAGTCCCACGATGACA |
| *Col1a1* | ACATGTTCAGCTTTGTGGACC | TAGGCCATTGTGTATGCAGC |
| *Col3a1* | TGGTAGAAAGGACACAGAGGC | TCCAACTTCACCCTTAGCACC |
| *Col4a1* | ACAAAAGGGTGATGCTGGAG | CTCCCTTTGTACCGTTGCAT |
| *Fn1* | TTAAGCTCACATGCCAGTGC | TCGTCATAGCACGTTGCTTC |
| *Postn* | TCACCGTTTCGCCTTCTTTA | CACCTTCAAAGAAATCCCCA |
